# Combined cellular and proteomics approach suggests differential processing of a native and a foreign vibrio in the sponge *Halicondria panicea*

**DOI:** 10.1128/mbio.01474-25

**Published:** 2025-06-27

**Authors:** Angela M. Marulanda-Gomez, Benjamin Mueller, Kristina Bayer, Mohammad Abukhalaf, Liam Cassidy, Andreas Tholey, Sebastian Fraune, Lucia Pita, Ute Hentschel

**Affiliations:** 1 Research Unit Marine Symbioses, GEOMAR Helmholtz Centre for Ocean Research Kiel 28402, Kiel, Germany; 2 Pelagic Microbiology, Institute of Biology and Chemistry of the Marine Environment (ICBM), Carl von Ossietzky Universität Oldenburg, Oldenburg, Germany; 3 Department for Freshwater and Marine Ecology, University of Amsterdam 1234, Amsterdam, the Netherlands; 4 Department for Marine Ecology, University of Bremen, Bremen, Germany; 5 Systematic Proteomics and Bioanalytics, Institute for Experimental Medicine, Christian-Albrechts-Universität Kiel 9179, Kiel, Germany; 6 Institute of Zoology and Organismic Interactions, Heinrich Heine Universität Düsseldorf, Düsseldorf, Germany; 7 Marine Biogeochemistry, Atmosphere and Climate group, Institut de Ciències del Mar–CSIC 58341, Barcelona, Spain; 8 Integrated Marine Ecology group (INMARE), Instituto de Investigaciones Marinas, Spanish Research Council, IIM-CSIC 83088 , Vigo, Spain; 9 Christian-Albrechts-Universität Kiel 9179, Kiel, Germany; University of Connecticut, Storrs, Connecticut, USA

**Keywords:** animal-microbe interactions, sponge-microbe symbiosis, phagocytosis, fluorescence-activated cell sorting (FACS), proteomics, sponge cells, *Vibrio* isolates, innate immunity, immune specificity

## Abstract

Phagocytosis is a conserved cellular mechanism for food uptake, defense, and animal-microbe interactions in metazoans. How the discrimination and subsequent processing of different microbes in marine invertebrates is facilitated remains largely unknown. Thereto, we combined a recently developed phagocytic assay with proteomics analysis to compare the phagocytic activity of the sponge *Halichondria panicea* upon encounter with the native Hal 281 (i.e., *H. panicea* isolate) and the foreign NJ 1 (i.e., *Nematostella vectensis* isolate) *Vibrio*. The sponge cell fraction was recovered after *Vibrio* exposure of 30 and 60 min and used for cellular (fluorescence-activated cell sorting and microscopy) and proteomics analyses. While the number of phagocytically active cells was similar between the isolates (*P* = 0.19), the distribution of vibrios over cell types differed (*P* = 0.02) over time, with the tendency for accumulation of NJ 1 in choanocyte-like cells compared to a shift of Hal 281 being incorporated from choanocyte-like to archaeocyte-like cells. Initially, both vibrios elicited a proteomic response related to bacterial infection and immunity (e.g., ADAM10, RAPTOR), followed by an increase of lysosomal and endocytic proteins (e.g., NPC2) after 60 min. The attenuation of the immune response and concomitant increase of vesicular trafficking in Hal 281 after 60 min corroborates cellular observations suggesting the fast transfer of Hal 281 from choanocyte-like cells to archaeocyte-like cells, compared to an accumulation of NJ 1 in the former. Subtle but distinct differences suggest strain-specific discrimination between the two tested vibrios and may indicate a degree of immune specificity in sponges.

**IMPORTANCE:**

Metazoans recognize and discriminate between different microbes. In marine invertebrates, the underlying mechanisms of microbial discrimination and immune specificity are, however, not well understood. Phagocytosis is a conserved cellular process from amoeba to humans that facilitates the ingestion and digestion of microbial cells and likely plays a role in this discrimination. To elucidate the molecular and cellular basis of this microbial discrimination, we examined the differential phagocytic processing of a native (i.e., sponge-isolated) and foreign (i.e., sea anemone-isolate) *Vibrio* in a marine sponge. Our findings revealed that both vibrios provoke an initial bacterial infection- and immune-related, followed by a lysosomal- and endocytic-related proteomic response. Nuanced differences in the cellular and molecular processing suggest a strain-specific discrimination between the two vibrios. This study investigates a mechanism for microbial discrimination in an early-divergent metazoan and may provide a valuable model for studying the evolution of immunity and its role in animal-microbe interactions.

## INTRODUCTION

“Modern-type phagocytosis”—characterized by actin-dependent engulfment and lysosome-mediated digestion—is thought to have initially emerged in early eukaryotic lineages as a mechanism for nutrient acquisition and resource reallocation (1, 2). In multicellular eukaryotes, it was subsequently adapted in specialized immune cells, such as macrophages, as a cell-defense mechanism to safeguard the organism from potentially hazardous agents (e.g., pathogens, damaged, senescent, or apoptotic cells) and to ensure its homeostasis (3–6). Phagocytosis is a cellular process for ingesting and digesting large particles (typically >0.5 µm in diameter), including microbes, foreign substances, and apoptotic cells, into cytosolic, membrane-bound vacuoles (i.e., phagosomes). This process comprises several subsequent steps including particle recognition via receptors, reorganization of the actin cytoskeleton for particle internalization, and intracellular digestion of the target particle through the activity of lytic enzymes (reviewed by references 6, 7). Bacterial pathogens often target or manipulate phagocytosis. For example, intracellular bacterial pathogens like *Legionella pneumophila* and *Mycobacterium tuberculosis* can circumvent host phagocytosis by evading ingestion, interfering with phagosome maturation, resisting degradation, and even escaping from the phagosome (8, 9). Interestingly, endosymbionts use analogous mechanisms to pathogens that aid in their colonization, acquisition, and maintenance within their host (10, 11).

Many metazoans form close relationships with symbiotic microorganisms, including archaea, bacteria, fungi, and viruses (12, 13). These relationships are often essential for the host organism by providing nutrients, stimulating development and growth, or protecting against pathogens (14–17). Evidence for the role of phagocytosis in microbe discrimination in endosymbiosis emerged from studies with a handful of metazoan model organisms (e.g., *Aiptasia* [anemone], *Euprymna scolopes* [squid], and *Acyrthosiphon pisum* [aphid]), for which the cultivation of symbionts, genetic manipulation, and/or implementation of cellular markers have been established (18–20). Microbial discrimination by the host may be of particular importance for bacterivore filter-feeding metazoans, such as corals, bivalves, or sponges, who in addition to discriminating between symbionts to retain and pathogens to eliminate, may have to recognize bacteria as food items.

The early branching phylum Porifera is an evolutionary and ecologically relevant group to study the role of phagocytosis in animal-microbe interactions. Sponges have no gut, and the same phagocytic cell may retain a function in defense and nutrition (4). As benthic filter feeders, sponges constantly remove bacteria from the surrounding water as food items (21–23). At the same time, sponges harbor distinct microbial communities inside their mesohyl at up to 2–3 orders of magnitude higher abundances than in the surrounding seawater (24–26). These symbionts support the sponge metabolism through various processes, including carbon and nitrogen cycling as well as the synthesis of vitamins and secondary metabolites (e.g., references 27–32). Recognizing and differentiating between food, symbiotic and/or pathogenic microbes is, therefore, paramount for these filter feeders. First observations of a reduced uptake of symbionts compared to seawater bacteria suggested underlying mechanisms for specific sponge-microbe interactions (33, 34). A recent study reported the differential expression of NLR-like receptors in response to food vs symbiont bacteria as a potential regulatory aspect of microbial discrimination in sponges (35), but little is still known on sponge-specific immune responses to microbes. Additional genomic studies further suggest that symbionts may avoid phagocytosis due to an enrichment of eukaryote-like proteins (e.g., ankyrins) presented on their surfaces (36–38), whereas seawater bacteria are taken up and digested. These proteins did promote bacteria persistence in cellular models, aiding the infection and survival of symbionts and pathogens in several eukaryotes, including amoeba, murine macrophages, and humans (39–41) by silencing innate immune responses. However, direct evidence if, and to what extent sponges facilitate bacterial discrimination on the cellular level is largely lacking.

A recently developed *in vivo* phagocytic assay applying cell dissociation followed by fluorescence-activated cell sorting (FACS) in the sponge *Halichondria panicea* may now help to bridge this gap (42). In the present study, this assay was used to assess whether the sponge *H. panicea* can differentiate between two vibrio strains: Hal 281 isolated from *H. panicea* (i.e., native) and NJ 1 isolated from *Nematostella vectensis* (i.e., foreign). The relative abundance of sponge phagocytic cells (i.e., with incorporated fluorescently labeled *Vibrio*) was quantified by FACS. Phagocytically active cells were additionally inspected using fluorescence microscopy to determine their size and morphological type. A corroborative proteomics analysis was performed to investigate underlying molecular mechanisms of cellular recognition, phagocytosis, and immunity upon microbial exposure. Therefore, the total number of differentially abundant proteins responding to bacterial exposure after 30 and 60 min was analyzed.

## MATERIALS AND METHODS

### Sponge collection

Specimens of the sponge *H. panicea* (Pallas, 1766) were collected at the Kiel fjord (54.329659 N, 10.149104 E; Kiel, Germany) at 1 m water depth in August 2023, cleaned from epibionts, trimmed to approximately equally sized fragments (volume: 32.3 ± 1.8 mL and wet weight: 5.9 ± 1.7 g [average ± S.D.]) containing 2–3 oscula, and left to heal and recover from collection on an *in situ* nursery at the collection site for 10 days (43). On the day of the experiment, individuals were brought to the climate-controlled aquarium facilities of GEOMAR Helmholtz Centre for Ocean Research (Kiel, Germany), placed in a semi-flow through aquarium system supplied with natural seawater pumped from the collection site, and left to acclimatize for 2 h at 18°C room temperature, 17°C water temperature, and a salinity of 16-17 PSU, closely resembling environmental conditions at the nursery.

### Bacteria preparation for the *in vivo* phagocytosis assay

The *Vibrio* isolates for the assay included the native sponge-associated isolate Hal 281 (isolated from *H. panicea*) and the foreign non-sponge-associated isolate NJ 1 (isolated from the sea anemone *Nematostella vectensis*). The similarity of 16S rRNA gene sequences between the two isolates was 95.62% (Table 1), and neighbor-joining phylogenetic analysis showed they belong to different clusters (Fig. S1A). *Vibrio* cultures were freshly grown before the day of the experiment in 100 mL liquid marine broth at 120 rpm, 25°C for 48 hours until the culture reached the mid to late exponential phase. The culture concentrations were estimated by OD_600_ and subsequently confirmed by flow cytometry measurements (final concentration approximately 4–6 × 10^8^ cells mL^−1^). The bacteria pellet was recovered by centrifugation (4,000 × *g* for 10 min), resuspended in filtered artificial seawater (FASW), and fluorescently stained with TAMRA (Thermo Fisher Scientific, C1171) the same day of the experiment (5 µM final concentration, for 90 min in the dark, at room temperature). After the excess dye was washed off by centrifugation (4,000 × *g* for 10 min), the bacteria were resuspended in FASW, and positive staining was confirmed with fluorescence microscopy (Fig. S1B and C). The *Vibrio* stocks were kept at 4°C, in FASW, protected from light, until the experiment took place (i.e., no longer than 30 min). Under these conditions, no significant bacterial growth is expected.

**TABLE 1:** Molecular and morphological comparison between the native and foreign *Vibrio* isolates Hal 281 and NJ 1, respectively^*a*^

Isolate	Host	Sequence length (bp) and GenBank accession number	Closest type strains	Similarity (%)	Average ± SD cell length (µm)	Average ± SD cell width (µm)
Hal 281	*Halichondria panicea*	1,517 (MT406665)	*Vibrio atlanticus* LMG 24300 (EF599163)/ *Vibrio cyclitrophicus* NBRC 107756 (AB682659)	99.47/99.80	1.98 ± 0.25	0.90 ± 0.08
NJ 1	*Nematostella vectensis* (anemone)	1,455 (PQ455196)	*Vibrio diazotrophicus* NBRC 103148 (BBJY01000042)/ *Vibrio plantisponsor* MSSRF60 (GQ352641)	99.40/99.37	2.39 ± 0.86	0.31 ± 0.14

^*a*^ Molecular analysis is based on 16S rRNA gene sequences. The two closest type strains, based on blastn analysis on 16S rRNA gene, are reported. The morphological features are reported as average ± standard deviation. GenBank accession numbers are given in parentheses.

### Phagocytosis assay

We followed a similar methodology and experimental approach as described in reference 42, a brief description of the method follows. Individual specimens of *H. panicea* were placed in incubators filled with unfiltered seawater (natural seawater bacteria concentration: ~10^5^ cells mL^−1^). Incubators with sponges were randomly assigned to one of the following treatments and incubated for either 30 or 60 min: addition of Hal 281 *Vibrio* (*n* = 5), addition of NJ 1 *Vibrio* (*n* = 5), or seawater control with no *Vibrio* addition (control, *n* = 5), (*n*: biological replicates per treatment and per timepoint). The 30 min incubation time was chosen to maximize incorporation into sponge cells (42), whereas the 60 min represents a compromise to have sufficiently high incorporations and at the same time allow more time for changes in the proteome to occur. The target concentration for the *Vibrio* treatments was 10^5^–10^6^ vibrios mL^−1^. Water samples for flow cytometry were taken throughout the incubation period to assess bacterial uptake by the sponges (Text S1; Table S1; Fig. S2).

### Sponge cell dissociation

The entire sponge individuals were recovered at the end of each incubation and used to extract the sponge cell fraction by a physical dissociation via differential centrifugation, as described in reference 42, (protocol adjusted after references 33, 44). Briefly, the entire sponge fragments were rinsed with sterile, ice-cold Ca- and Mg-free artificial seawater (CMFASW) (45), cut into small fragments, and incubated for 15 min in ice-cold CMFASW with EDTA (25 mM) on a shaker. Samples were filtered through a cell strainer (40 µm), and the homogenate was centrifuged at 500 × *g*, for 5 min at 4°C. The supernatant was discarded, and the sponge cell pellet was resuspended in 4 mL of ice-cold CMFASW. Aliquots (1 mL) of the cell suspension were either sampled for proteomics analysis (see below) or fixed with PFA (final concentration 4%) overnight for estimating phagocytosis using FACS.

### Estimation of *Vibrio* phagocytosis by sponge cells

Phagocytosis of the *Vibrio* isolates by sponge cells was estimated using fluorescence-activated cell sorting (FACS) following the protocol by (42). Shortly, the fixative was washed off the cells by centrifugation (500 × *g*, for 5 min at 4°C), and the pellet was resuspended in 1 mL of ice-cold CMFASW. The concentration of the dissociated sponge cells was adjusted to approximately 3 × 10^7^ cells mL^−1^. Prior to the analysis, sponge cells were filtered again through a cell strainer (40 µm), and their nuclei stained with DAPI (final concentration 0.7 ng µL^−1^) to detect the “bulk” sponge cells population based on the dye fluorescence (355 nm UV laser and filter 448/59 nm). FACS analysis was performed on a MoFlo Astrios EQ cell sorter (Beckman Coulter) using the Summit software (v6.3.1). Each sample was run five times, and a total of 500 k events were recorded for each technical replicate. The side scatter (SSC), DAPI, and the TAMRA-stained bacteria fluorescence (laser 561 nm and filter 692/75 nm) were used to identify and quantify the sponge cells that had incorporated the *Vibrio* isolates (40). The control sponges (i.e., individuals incubated without isolates) were used to correct for events corresponding to natural auto-fluorescence in the cells (as in reference 42). We define sponge phagocytic cells as those that had incorporated the fluorescent vibrios during the assay, whereas non-phagocytic cells as those without incorporated vibrios (i.e., according to the presence or lack of fluorescence signal in the TAMRA channel, respectively). The relative (%) phagocytic and non-phagocytic cell fraction was estimated in relation to the total number of events from these two gates (for details, see reference 42). To test the effect of the *Vibrio* type (i.e., native Hal 281 vs foreign NJ 1) and of incubation time (i.e., 30 vs 60 min), a two-way ANOVA analysis was performed (significance was determined at α = 0.05). Statistical analysis was performed in R-studio (V4.2.1; Rstudio Team 2022) by fitting an analysis of variance model [aov () function].

### Determination of phagocytic sponge cells using fluorescence microscopy

**Fig 1:**
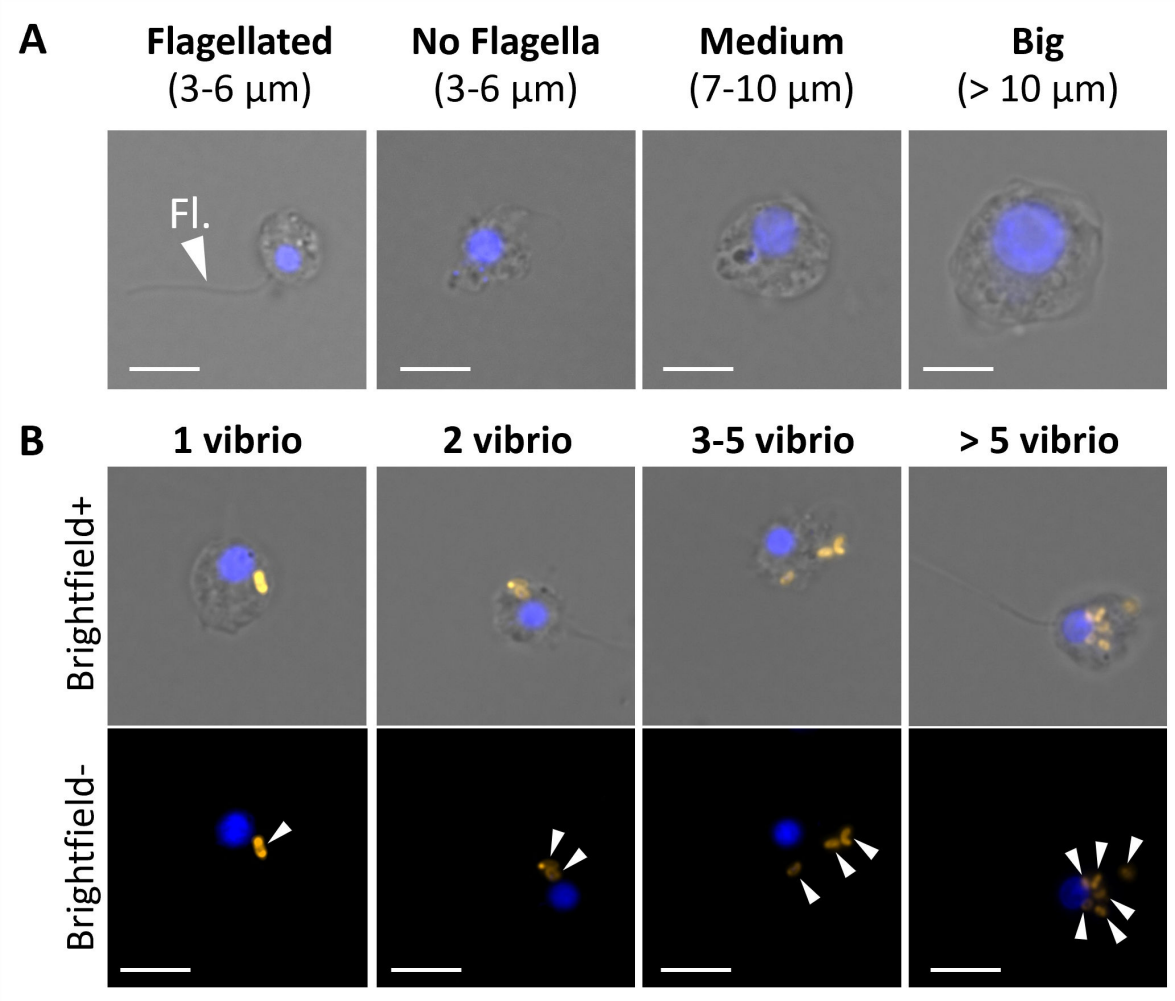
Phagocytic cell types of *H. panicea* individuals exposed to *Vibrio* isolates based on fluorescence microscopy. Representative fluorescent microscopy pictures showing (**A**) different phagocytic cell categories and (**B**) different amounts of *Vibrio* cells incorporated into sponge cells. Scale bars: 5 µm. Sponge cell nuclei (blue) stained with DAPI. Fl, flagella. Arrowheads, TAMRA-stained *Vibrio*.

Dissociated sponge cell suspensions of three random individuals per treatment were sub-sampled and inspected by fluorescence microscopy to determine the cell types involved in phagocytosis of Hal 281 and NJ 1. A total of 30 phagocytic cells per individual were counted (30 phagocytic cells × 3 replicates = 90 cells per treatment and timepoint), and the size of the cells as well as the number of TAMRA-stained *Vibrio* cells incorporated per cell was recorded. Cells were mounted on microscopy slides using ROTI mount FluorCare DAPI and examined under an inverted fluorescence microscope equipped with a camera (Axio Observer Z1 with Axiocam 506 and HXP-120 light; Zeiss), at a total magnification of 100×, using the filters 49 DAPI (335–383 nm for sponge nuclei) and 43 HE DsRed (538–562 nm for TAMRA-stained *Vibrio* sp.). ZEN Blue Edition software (Zeiss) was used for acquiring and editing pictures. Sponge cells were morphologically classified into four categories (Fig. 1A): (i) small-sized flagellated cells (3–6 µm Ø), (ii) small-sized cells with no visible flagella (3–6 µm Ø), (iii) medium-sized cells (7–10 µm Ø), and (iv) big cells (>10 µm Ø). As sponge cells change their type-specific morphological shape due to the dissociation process, we could only classify the cells based on their size and the presence/absence of a flagellum. We further estimated the number of TAMRA-stained *Vibrio* cells that were incorporated per observed sponge phagocytic cell. The number of vibrios per sponge cell was divided into four categories (Fig. 1B): 1 vibrio, 2 vibrios, 3–5 vibrios, >5 vibrios. One-way ANOVAs were run per treatment condition (i.e., per *Vibrio* type and per incubation time separately) to test if the cell types involved in the bacteria incorporation as well as the number of *Vibrio* cells incorporated per phagocytic cell differ between the *Vibrio* isolates presented to *H. panicea*. Additionally, a PERMANOVA [adonis2 () function], package vegan in R-studio) was performed in the combined data set to test if the distribution of phagocytic cell types as well as the number of vibrios incorporated per cell changed between *Vibrio* isolates and over time.

### Identification of differentially abundant proteins using proteomic analysis

Aliquots (1 mL) from the dissociated sponge cell suspensions were washed by centrifugation (500 × *g*, for 5 min at 4°C), the supernatant was discarded, and the cell pellet was resuspended in 1 mL of lysis buffer (5 M urea, 1% Triton x-100, 5 mmol L^−1^ dithiothreitol, 1× cOmplete mini EDTA-free, and 50 mM Tris; pH 7.5–8). Samples were thoroughly vortexed for 30 s and incubated in a shaker (1,500 rpm) for 40 min at 37°C. Throughout this step, samples were vortexed every 10 min for 30 s. Subsequently, they were placed in an ultrasonic bath for 5 min. Debris was pelleted by centrifugation (20,000 × *g*, for 30 min at 10°C), and the supernatant was transferred into a new tube and stored at −80°C until further processing. Protein concentrations were determined using the Pierce BCA Protein assay kit (Thermo) according to manufacturer’s instructions. Fifty micrograms of protein of each sample was processed further according to the SP3 protocol (46) as follows. Proteins were reduced by the addition of Dithiothreitol (DTT; 10 mM final concentration) and mixing at 1,000 rpm, 56°C for 30 min. Alkylation was performed by the addition of chloroacetamide (50 mM final concentration) and mixing at 1,000 rpm, 25°C for 20 min. Fifty microliters of a mixture of hydrophobic and hydrophilic SP3 beads (20 µg µL^−1^) was added, reaching a 20:1 (beads:protein) ratio. Binding to beads was induced by adding ethanol to 54% and then mixing at 1,500 rpm, 25°C for 15 min. Beads were washed three times, each washing step consisted of (i) centrifugation (21,000 × *g*, 25°C for 2 min), (ii) binding beads to a magnet and discarding the supernatant, (iii) adding 400 µL of 80% ethanol and ultrasonicating until beads disperse, and (iv) finally vortexing for 10 s. The remaining supernatant was discarded after the last washing step, and beads were reconstituted in 100 µL of triethyl ammonium bicarbonate (100 mM). Proteins were digested at 37°C overnight with 0.7 µg trypsin/Lys-C mix (Promega). Afterward, supernatant-containing peptides were vacuum dried and then reconstituted in 100 µL 3% acetonitrile (ACN) and 0.1% trifluoroacetic acid (TFA). An additional desalting step was performed using Pierce C18 100 µL pipette tips as follows. Tips were washed twice with 100 µL (50% ACN) and equilibrated twice with 100 µL (3% ACN, 0.1% TFA). Peptides were loaded by pipetting up and down 10× and then washed 2× with 100 µL (3% ACN, 0.1% TFA). A first peptides elution was done with 100 µL 50% ACN and 0.1% TFA, and a final elution with 50 µL 70% ACN, 0.1% TFA. Eluted peptides were vacuum dried, reconstituted in 3% ACN, 0.1% TFA, and analyzed by liquid chromatography-mass spectrometry (LC-MS). Chromatographic separation was performed on a Dionex U3000 nano HPLC system equipped with an Acclaim pepmap100 C18 column (2 µm particle size, 75 µm × 500 mm) coupled online to a mass spectrometer. The eluents used were eluent A (0.05% formic acid [FA]) and eluent B (80% ACN + 0.04% FA). The separation was performed over a programmed 132 min run. Initial chromatographic conditions were 4% B for 2 min followed by linear gradients from 4% to 50% B over 100 min, then 50% to 90% B over 5 min, and 10 min at 90% B. Subsequently, an inter-run equilibration of the column was achieved by 15 min at 4% B. A constant flow rate of 300 nL min^−1^ was employed. Two technical replicates of each sample were analyzed, and wash runs were performed between samples. Data acquisition following separation was performed on a QExactive Plus (Thermo). Full scan MS spectra were acquired (300–1,300 *m z*^−1^, resolution 70,000), and subsequent data-dependent MS/MS scans were collected for the 15 most intense ions (Top15) via HCD activation at NCE 27.5 (resolution 17,500, isolation window 3.0 *m z*^−1^). Dynamic exclusion (20 s duration) and a lock mass (445.120025) were enabled.

MS raw data were analyzed against *H. panicea* proteome (PRJEB69565; generated by the Aquatic Symbiosis Genomics Project) built from Baker3 genome models (https://github.com/Aquatic-Symbiosis-Genomics-Project/sponge_annotations/tree/main/results/odHalPani1) on the chromosome-level genome assembly (18,233 sequences; accessed 24 January 2025), and known contaminants (cRAP) using the CHIMERYS identification search engine linked to Proteome Discoverer 3. 1.1.9 (Thermo). The raw data files can be found in the ProteomeXchange repository (identifier PXD063400) (47). Enzyme was set to trypsin with two missed cleavages tolerance. 20 ppm fragment ion mass errors were tolerated. Carbamidomethylation of cysteine was set as a fixed modification, and oxidation of methionine (M) was tolerated as a variable modification. Strict parsimony criteria were applied, filtering peptides and proteins at a 1% false discovery rate (FDR). Label-free quantification method based on the intensities of the precursor ions was used. Proteins were filtered to have “high” FDR combined confidence and at least two identified peptides. Data were further analyzed by Excel and Perseus v 1.6.15.0 (48). Protein intensities were averaged for technical replicates and then normalized by median-based normalization. Log2 transformed intensities were grouped into six groups depending on the *Vibrio* treatment and incubation time (each with four biological replicates) and filtered to contain four values in at least one group. Missing values were imputed from a normal distribution separately for each replicate (width 0.3, downshift 1.8). Statistical analysis was done using ANOVA, permutation-based FDR of 0.05 or 0.01, as indicated. Furthermore, we performed a direct pathway analysis (directPA R package) (49) on the quantified proteins based on their log2 fold changes for each treatment relative to the control to better understand the sponge cells’ protein dynamics upon exposure to each *Vibrio* treatment. This analysis allowed us to identify proteins with higher or lower abundances in both treatments relative to the control. The function returns a list of proteins and probabilities based on the direction of change. Proteins with a high probability (*P*-value < 0.01) were filtered for each direction, and their role in different pathways was further characterized (see “Functional annotation of proteins”).

### Functional annotation of proteins

Identified protein sequences from *H. panicea* were annotated to UniProt identifiers of *Amphimedon queenslandica* proteome (Uniprot UP000007879_444682) by blast search (at protein level, *e*-value < 1e−5; Galaxy 24.2.4c1 [50]) and to KO by BlastKOALA using the Eukaryotes KEGG gene database (51). The best Blastp matches with *A. queenslandica* were used as input in the DAVID web server (knowledgebase v2024q1) (52, 53) to perform Gene Ontology (GO) functional annotation and enrichment analysis of the significantly differentially abundant proteins (DAPs). GO terms and fold enrichment values were analyzed in REVIGO (v1.8.1 [54]) to reduce redundancy and group them based on semantic similarity (SimRel).

The groups of proteins obtained from the directional analysis were further characterized to identify proteins likely to be involved in endocytic-, phagocytic-, and lysosomal-based pathways (from now on referred to as vesicle trafficking-related pathways), as well as bacterial infection, and/or immunity pathways based on KEGG annotations. KEGG pathways were reconstructed with the KEGG Mapper Reconstruct web tool (v5) (51, 55). The KEGG identifier numbers from the BlastKOALA annotation were used as input for reconstructing the pathways. KEGG pathways used for the protein classification included endocytosis (map04144), lysosome (map04142), phagosome (map04145), autophagy and mitophagy (map04140, map04138, map 04136, map 04137), cytoskeletal motor proteins (map04814) (these five categories correspond to vesicle trafficking-related pathways), bacterial infection (map05110, map05120, map05130, map05131, map05132, map05133, map05134, map05135, map05152), and immunity (map04623, map04625, map04657, map04611, map04612, map04613, map04620, map04621, map04622, map04624, map04666).

## RESULTS

### Phagocytically active cell fraction after exposure to a native and a foreign *Vibrio* isolate

**Fig 2:**
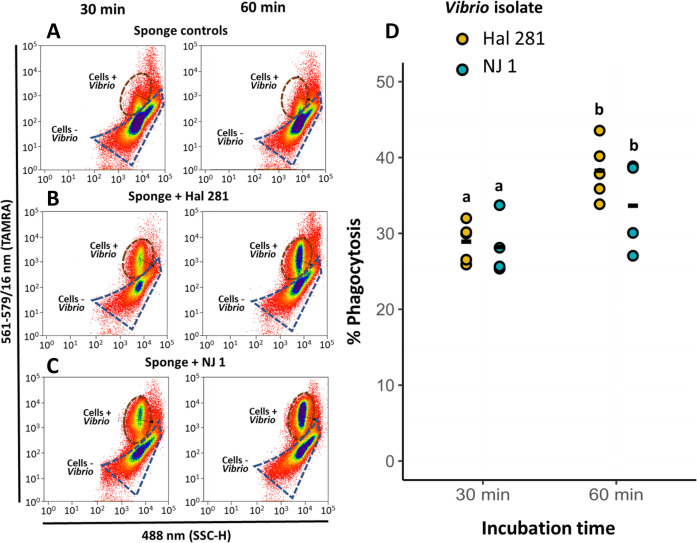
Fluorescence-activated cell sorting (FACS) analyses estimates of phagocytic cells. Representative cytograms for (**A**) sponge controls (incubated without *Vibrio* addition) and sponges incubated with the (**B**) native Hal 281 and (**C**) foreign NJ 1 *Vibrio* isolates, for 30 min (left) and 60 min (right). Brown and blue dashed outlines: Gates of phagocytic cells with (+) or without (–) incorporation of *Vibrio*, respectively. A total of 500k events were recorded in each case. (**D**) Estimates of phagocytically active sponge cells. The relative number (%) of cells phagocytizing *Vibrio* cells (+TAMRA signal) to the total of sponge cells (DAPI-stained, SSC-H signal) was estimated via FACS analysis (whole data set in Table S2). Bold line: average for the 4–5 biological replicates. Treatments marked with different letters are significantly different at ɑ = 0.05.

The average (±SD) number of sponge cells recovered after dissociation was approximately 4.89 ± 3.00 × 10^7^ cells mL^−1^. The population of phagocytic cells of the *H. panicea* individuals incubated with the *Vibrio* isolates was clearly distinct from the corresponding gate in the control sponges (i.e., individuals incubated without isolates) (Fig. 2A through C). In the 30 min assays, the percentage of phagocytic cells was on average 28.9% ± 2.6% for Hal 281 and 28.2% ± 3.9% for NJ 1 (Fig. 2D; Table S2) with no significant difference between the isolates (one-way ANOVA, *F* = 6.1, *P* = 0.99; df = 3). In the 60 min assays, the average percentage of phagocytic sponge cells was 38.3% ± 3.8% for Hal 281 and 33.6% ± 6.0% for NJ 1. These percentages represent, on average, a 9.3% and 5.4% increase for Hal 281 and NJ 1, respectively, compared to the estimates for the 30 min assays (Fig. 2D; Table S2). There was no effect of the *Vibrio* isolate on the phagocytic activity of *H. panicea* cells (two-way ANOVA, *F* = 1.9, *P* = 0.19; df = 1; Fig. 2D), whereas incubation time significantly increased the number of bacteria incorporated into the sponge cells (two-way ANOVA, *F* = 15.9, *P* = 0.001; df = 1; Fig. 2D).

### Distribution of *Vibrio* cells in different phagocytic cell types

**Fig 3:**
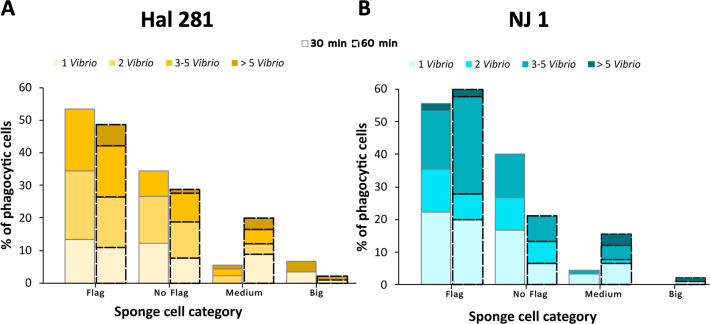
Distribution of *Vibrio* cells in the different phagocytic cell types after 30 min (gray outlined bars) and 60 min (dashed bars) incubations with the (**A**) native isolate Hal 281 and (**B**) foreign isolate NJ 1 based on microscopy cell counts. Flagellated (Flag) and no flagellated (No Flag): 3–6 µm Ø; Medium: 7–10 µm Ø; Big: >10 µm Ø.

Fluorescence microscopy observations of phagocytic cells (90 cells per treatment and time point) showed that *Vibrio* phagocytosis was performed by sponge cells with different sizes and morphologies (Fig. 1). In both *Vibrio* treatments, approximately 88% and 96% of the total phagocytic cells observed in the 30 min assays comprised small-flagellated cells (53%–55%) and cells with no visible flagella (34%–40%) (3–6 µm Ø. Figure 3; Fig. S3; Table S3). Medium (7–10 µm Ø) and big cells (>10 µm Ø) only represented approximately 4%–12% (i.e., 4–11 cells) of the recorded cells (one-way ANOVA, *F* = 18.6, *P* < 0.001, df = 7; Fig. S3). After the 60 min assays, the small-flagellated cells continued to be significantly higher than the other cell types (one-way ANOVA, *F* = 28.3, *P* < 0.001, df = 7; Fig. S3). When combining the two data sets, a significant interaction between time (30 vs. 60 min) and *Vibrio* isolate (Hal 281 *vs*. NJ 1) on the distribution of cell types involved in phagocytosis was revealed explaining 24% of the variation in the data (PERMANOVA, *F* = 7.8, *P* = 0.003, df = 1; Fig. 3; Table S4A). Thus, the effect of time on phagocytic cell type distribution differed between the two *Vibrio* isolates. The percentage of flagellated cells and non-flagellated cells appeared to decrease from 30 to 60 min for both isolates, yet this decline was not significant (*t*-test flagellated: *t* = 0.61 and −1.39, *P* = 0.60 and 0.30, df = 2; non-flagellated: *t* = 0.49 and 2.36, *P* = 0.67 and 0.14, df = 2, Hal 281 and NJ 1, respectively). Similarly, the medium-size cells tended to increase after the 60 min incubation for both Hal 281 and NJ 1 treated sponges, but also not significantly (*t*-test, *t* = −1.66 and −2.77, *P* = 0.24 and 0.11, df = 2, respectively).

We further estimated the amount of *Vibrio* cells that were incorporated per phagocytic sponge cell (Fig. S4; Table S3). Most sponge cells (88%–98%) incorporated a maximum of 5 vibrio cells after both 30 and 60 min incubations (Fig. 3). The number of vibrios per sponge cell was not affected by incubation time but by *Vibrio* isolate (PERMANOVA, *F* = 2.9, *P* = 0.03, df = 1, Fig. 3; Table S4B). The percentage of sponge cells with >3 vibrios was higher in sponges incubated with the foreign *Vibrio* NJ 1 than in sponges incubated with the native isolate Hal 281. Additionally, the distribution of *Vibrio* cells over the different types of phagocytic cells was significantly different between Hal 281 and NJ 1, and this effect of the *Vibrio* isolate explains 19% of the variation in the data (PERMANOVA, *F* = 2.5, *P* = 0.02, df = 1, Fig. 3; Table S4C). The difference was especially evident in the flagellated cells of sponges incubated with NJ 1, which incorporated a higher number of vibrios than sponges incubated with Hal 281. Exposure time did not affect the distribution of vibrios among cell types.

### Differentially abundant protein profiles upon *Vibrio* and seawater bacteria exposure

In total, 4,585 and 4,690 proteins were quantified on sponge cell fractions after 30 and 60 min incubations, respectively (see whole data set in ProteomeXchange repository identifier PXD063400). The principal component analysis (PCA) revealed a distinct proteome signature of the NJ 1 treated sponges, which formed a separate cluster from control and Hal 281 in both 30 min (PERMANOVA, *F* = 1.79, *P* = 0.016, df = 2) and 60 min (PERMANOVA, *F* = 2.47, *P* = 0.001, df = 2) incubation data sets (Fig. S5A and B). Moreover, an effect of Hal 281 exposure on the sponge cells proteome was detected after 60 min incubations (Fig. S5A and B). Thus, 60 min exposure to the *Vibrio* isolates had the most prominent effect on the proteome of *H. panicea* cells.

**Fig 4:**
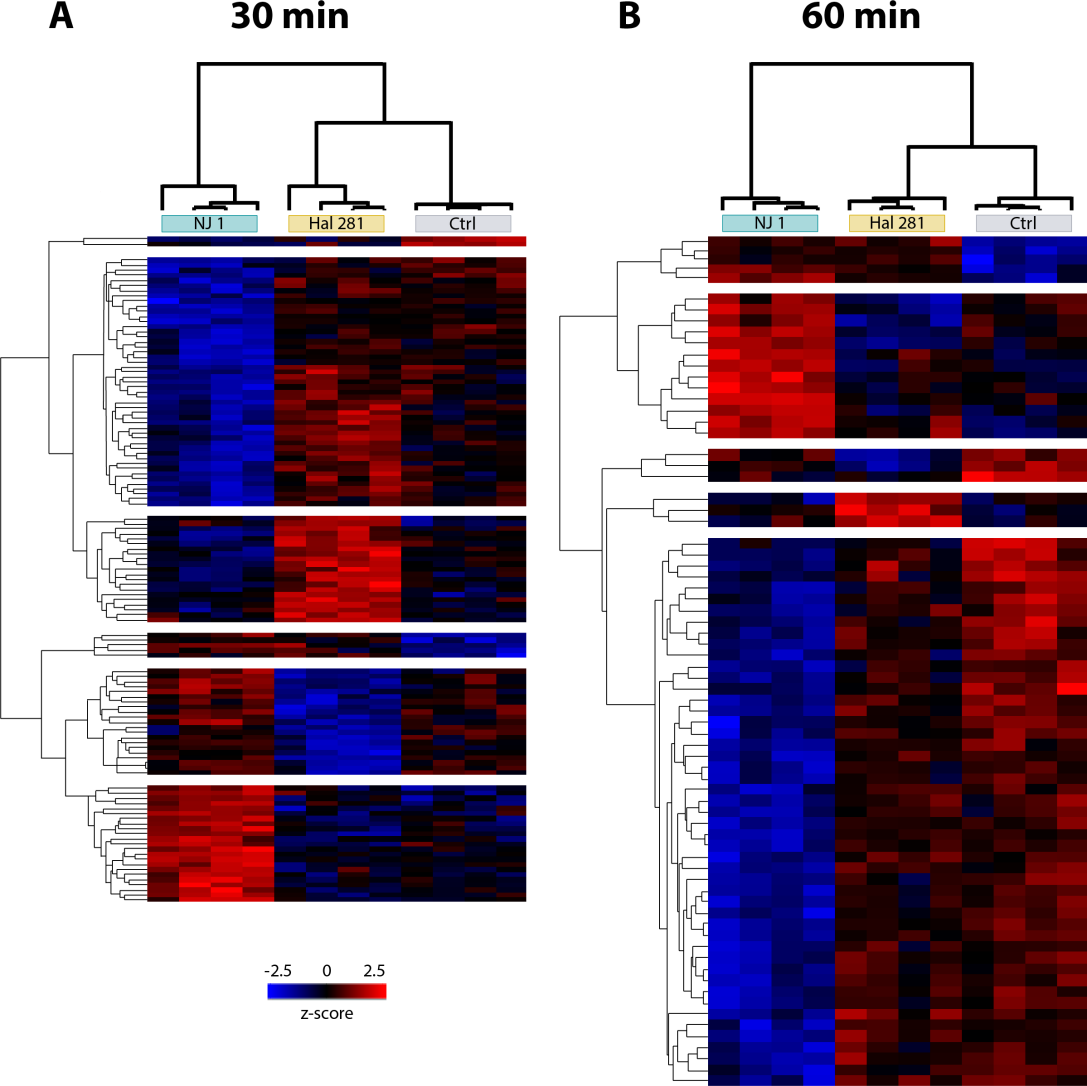
Heatmaps of differentially abundant proteins (DAPs) in *H. panicea* cells after (**A**) 30 min and (**B**) 60 min sponge incubations with the native and foreign *Vibrio* isolates Hal 281 and NJ 1, respectively. Sponges incubated without the addition of *Vibrio* isolates served as controls (Ctrl). Proteins were defined as differentially abundant relative to all the treatment groups with ANOVA permutated-based FDR *P*-value < 0.05, *n* = 4. Each row represents one protein, and each column one sponge sample. Dendrograms show protein clusters with similar abundance based on Euclidean distance and normalized *z*-score. Significant clusters are indicated by breaks in the heatmap.

We identified a total of 73 and 121 significant differentially abundant proteins (DAPs) between all the treatment groups after the 30 and 60 min incubations, respectively (ANOVA, permutation-based FDR = 0.05, Table S5; Fig. S5C and D). BlastKOALA provided annotation for approximately 60%–74% of the total quantified DAPs at both time points (Fig. S5C and D. The full annotation report can be found in Table S5. The DAP profile of sponges exposed to Hal 281 exhibited more similarity to the control at both time points, contrary to NJ 1 (Fig. 4A and B; Fig. S5A and B). At 30 min, a total of 49 DAPs were shared between the Hal 281 treated sponges and the control, whereas specimens exposed to NJ 1 no DAPs were shared with control sponges (Fig. S5C and D). After 60 min incubations, the total number of DAPs increased, but Hal 281 and control continued to share more DAPs than NJ 1 and control (49 and 21 proteins, respectively. Figure S5C and D). Based on the abundance profile of DAPs within each sample, biological replicates were clustered according to treatment (Fig. 4A and B, upper dendrograms). Moreover, DAPs clustered into six and five different clusters based on their abundance profile similarities after 30 and 60 min, respectively (left dendrograms Fig. 4A and B).

### Protein-related functions of *Vibrio* exposure relative to seawater control

**Fig 5:**
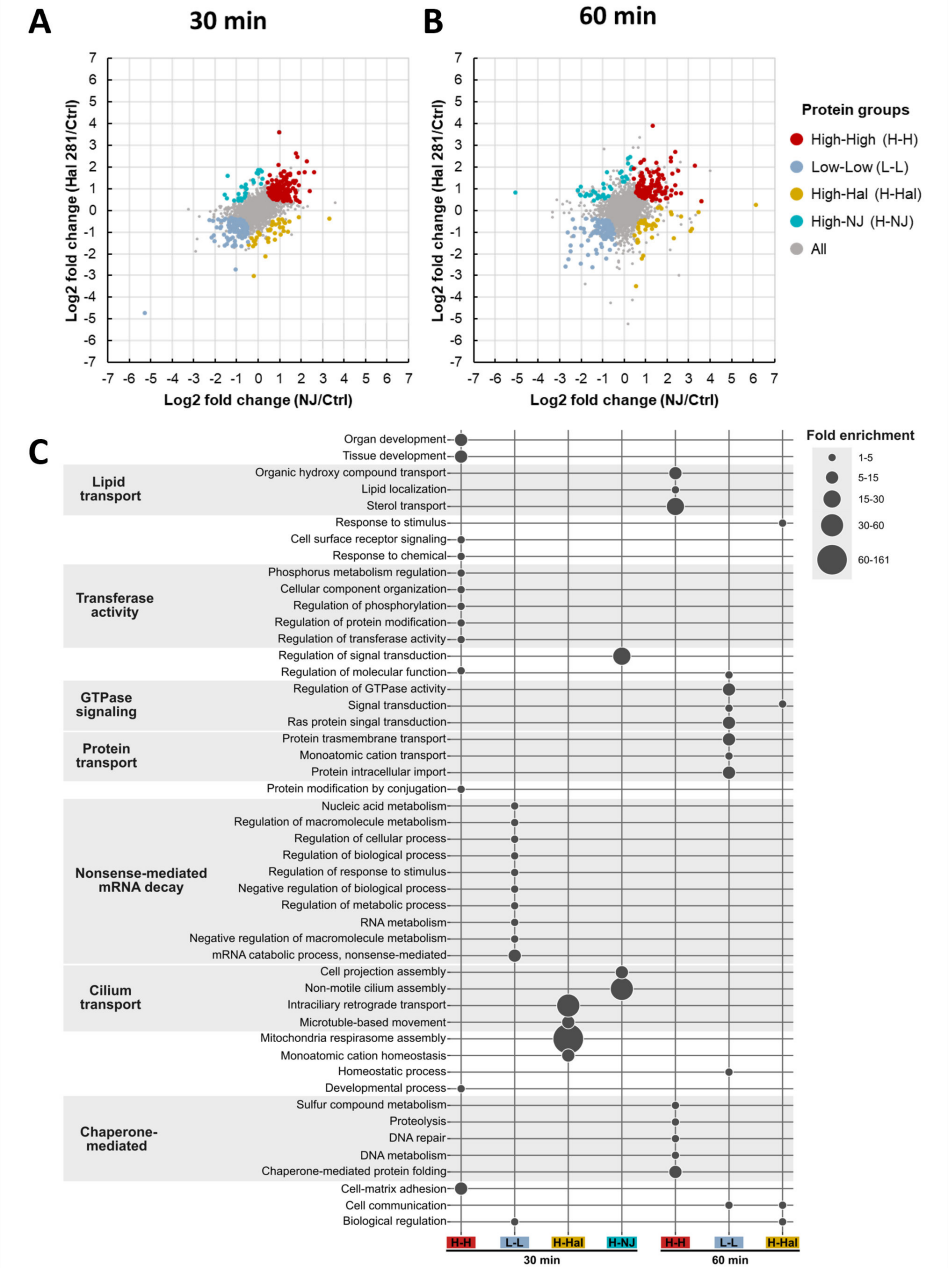
Directional pathway analysis of proteins identified after incubating *H. panicea* with a sponge-associated (Hal 281) and a foreign (NJ 1) *Vibrio* isolate for (**A**) 30 min and (**B**) 60 min. Individuals incubated without the addition of *Vibrio* isolate served as control (*n* = 4 biological replicates per treatment). Proteins with higher or lower abundances in both treatments relative to the control (Ctrl) using protein fold changes and with a high probability (*P*-value < 0.01) are shown. (**C**) Predicted functions of differentially abundant proteins that were high or low in *H. panicea* cells after exposure of 30 min and 60 min to the native and foreign *Vibrio* isolates Hal 281 and NJ 1, respectively. Functions are based on GO annotations assigned by DAVID as biological processes. Functional descriptions correspond to REVIGO semantic clustering. Circle size indicates the fold enrichment value of each function. This bubble plot is equivalent to the tree maps generated by REVIGO (dispensability threshold < 0.5). Only groups with more than 30 proteins with GO annotations are shown in the plot. H-H and L-L: proteins were high and low in response to both *Vibrio* isolates, respectively. H-Hal: proteins were high in Hal 281 treated sponges but low or unchanged in NJ 1 exposed individuals. H- NJ: proteins were high in NJ 1 treated sponges but low or unchanged in Hal 281 exposed individuals.

We further performed a direction pathway analysis (directPA) (49) on the quantified proteins based on the log2 fold changes of each treatment relative to the control to better understand the protein dynamics elicited by the two *Vibrio* strains. According to this analysis, we defined four DAP categories: high or low abundance in both *Vibrio* treatments (H-H and L-L, respectively), high abundance in Hal 281 but low or unchanged abundance in NJ 1 (H-Hal), and high abundance in NJ 1 but low or unchanged abundance in Hal 281 (H-NJ) (Fig. 5A and B; Table S6). The two former categories (H-H and L-L) represent a general *Vibrio* response (in comparison to a mixed seawater consortium, i.e., our control), while the latter two categories (H-Hal and H-NJ) represent a *Vibrio* strain-specific proteomic responses. At 30 min, 201 and 176 proteins were identified in the H-H group and the L-L group, respectively (Fig. 5A and B; Table S6). In addition, the abundance of 31 proteins was only increased in response to Hal 281 (H-Hal), whereas 42 proteins exclusively increased in response to NJ 1 (H-NJ) (Fig. 5A and B; Table S6). At 60 min, the number of proteins observed to be high (H-H) and low (L-L) in *Vibrio*-treated individuals decreased to 175 and 130 proteins, respectively (Fig. 5A and B; Table S6). Conversely, the proteins with different abundances in each specific *Vibrio* treatment increased to 65 and 60 proteins for the H-Hal and H-NJ groups, respectively (Fig. 5A and B; Table S6). These results indicate an increase in *Vibrio* strain-specific changes in the proteome over time.

Gene Ontology (GO) enrichment analysis of proteins belonging to the four DAP categories revealed no significant enrichment (FDR corrected *P*-value < 0.05). Yet, we used the GO annotation (Table S7) to predict the functions in the DAP categories. After the 30 min incubation, *H. panicea* sponge response to both *Vibrio* isolates consisted mainly of an increased abundance (H-H category; 165 annotated DAPs) of proteins involved in the regulation of transferase activity (GO:0051338) such as protein modification (GO:0031399) and phosphorylation (GO:0042325 and GO:0051174), and tissue development (GO:0009888 and GO:0048513), and a decrease (L-L; 123 annotated DAPs) of those associated with nonsense-mediated decay pathway for nuclear-transcribed mRNA catabolic process (GO:0000184) and, overall, with the regulation of metabolic and cellular processes, as well as response to stimulus (GO:0048583) (Fig. 5C). The *Vibrio* strain-specific response was characterized by an increase in proteins related to mitochondrial respirasome assembly (GO:0097250, GO:0007018, and GO:0035721) upon exposure to the native Hal 281 strain (H-Hal; 23 annotated DAPs), whereas the foreign NJ 1 (H-NJ; 36 annotated DAPs) strain increased the abundance of DAPs involved in the regulation of signal transduction (GO:0009966) and cilium assembly (GO:1905515 and GO:0030031) (Fig. 5C).

The proteomic response of sponges after 60 min incubation with one of the two *Vibrio* types showed an increased abundance (H-H; 132 annotated DAPs) in proteins related to chaperone-mediated protein folding (GO:0061077, GO:0006259, GO:0006281, GO:0006508, and GO:0006790) and sterol transport (GO:0015918, GO:0010876, and GO:0015850). At the same time, a decreased abundance (L-L; 102 annotated DAPs) in proteins associated with Ras protein signal transduction (GO:0007265, GO:0007165, GO:0023052, GO:0043547, and GO:0065009), protein transmembrane import into intracellular organelles (GO:0044743, GO:0006812, GO:0055085, and GO:0071806), homeostasis processes (GO:0042592 and GO:0055080), and cell communication (GO:0007154) was recorded (Fig. 5C). Apart from these general *Vibrio*-specific responses, Hal 281 treatment increased the abundance of proteins (H-Hal; 30 annotated DAPs) associated with signal transduction (GO:0007165, GO:0023052), response to stimulus (GO:0051716 and GO:0050896), cell communication (GO:0007154), and biological regulation (GO:0065007) (Fig. 5C). None of the proteins increasing upon 60 min exposure to the foreign isolate NJ 1 (H-NJ; 35 annotated DAPs) was associated with GO biological processes from our analysis in DAVID and was, therefore, not included in Fig. 5C.

### Proteins associated with vesicle trafficking, bacterial infection, and immunity upon *Vibrio* exposure

**Fig 6:**
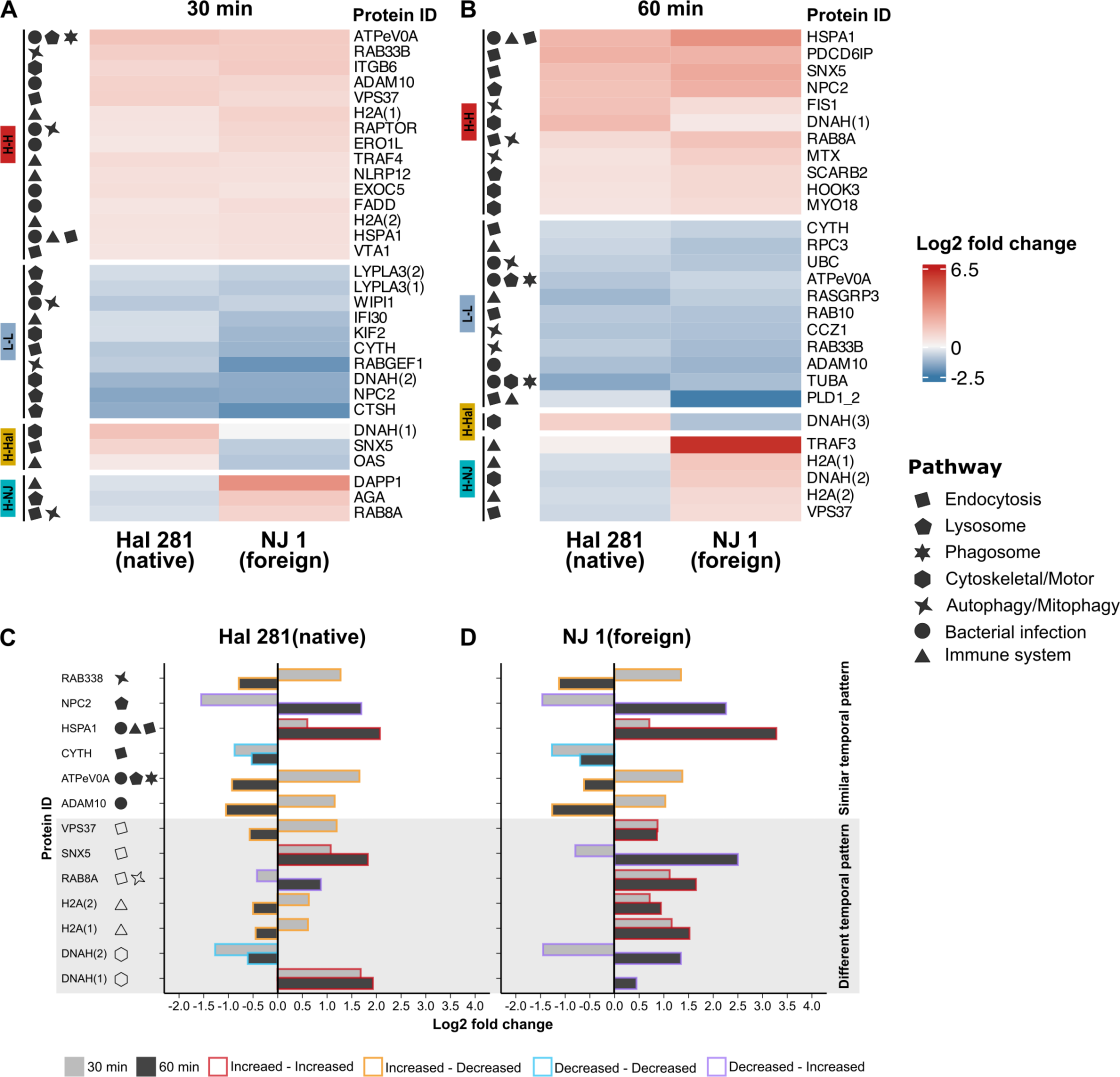
Predicted significantly differentially abundant proteins (DAPs) involved in pathways related to vesicle trafficking, bacterial infection, and immunity after (**A**) 30 min and (**B**) 60 min sponge incubations. Annotation is based on KEGG terms of the DAPs identified in *H. panicea* cells after exposure to the native and foreign *Vibrio* isolates Hal 281 and NJ 1, respectively. Color gradient: protein abundance log_2_ fold change relative to the control treatment. Each row represents one protein, and each column one sponge sample. Significant protein clusters with similar abundance are indicated by breaks in the heatmaps. Numbers in brackets indicate different proteins with the same annotation. Temporal dynamics (C) of the 13 DAPs present at 30 min (light gray bars) and 60 min (dark gray bars) upon exposure to the (**A**) native and (**B**) foreign *Vibrio* isolates. Colored outlines represent: increased abundance at both time points (I-I; red), decreased abundance at both time points (D-D; blue), increased followed by decreased abundance (I-D; orange), and decreased followed by increased abundance (D-I; purple). KEGG pathways included vesicle trafficking (endocytosis [map04144], lysosome [map04142], and phagosome [map04145]), bacterial infection (map05110, map05120, map05130, map05131, map05132, map05133, map05134, map05135, map05152), and immunity (map04623, map04625, map04657, map04611, map04612, map04613, map04620, map04621, map04622, map04624, map04666).

The groups of proteins obtained from the directional analysis were further characterized to identify proteins associated with vesicle trafficking (i.e., endocytosis, lysosome, phagosome, cytoskeletal/motor, and autophagy/mitophagy), bacterial infection, and/or immunity pathways based on KEGG annotation (see Material and Methods for details and Table S8). At 30 min, a total of 31 proteins were classified under these KEGG terms, and 15 of these exhibited high abundance profiles (H-H), whereas 10 showed low abundances (L-L) in response to both *Vibrio* isolates, compared to the control treatment (Fig. 6A). Among the proteins in the H-H group, we identified proteins related to bacterial infection and immunity pathways. Among them, a transmembrane metalloproteinase (ADAM10; K02154), a regulatory-associated protein of the mTOR signaling pathway (RAPTOR; K07204), an exocyst complex component (EXOC5; K19984), an associated death domain protein (FADD; K02373), and an endoplasmic reticulum oxidoreductase (ERO1L; K10950) (Fig. 6A). We also detected endocytic- (ko04144), phagosome-related (ko04145) and lysosome-related (ko04142) proteins in the H-H group, such as a heat shock protein 70 (HSPA1; K03283), a vacuolar ATP synthase (ATPeV0A; K02154), and two vacuolar proteins: one a member of the endosomal sorting complex required for transport system (VPS37; K12185) and the other involved in the endosomal multivesicular bodies pathway (VTA1; K12199) (Fig. 6A). Among the proteins in the L-L group, we found four proteins from the lysosomal pathway: two lysophospholipases (LYPLA3; K06129), a cathepsin (CTSH; K01366), and an intracellular cholesterol transporter (NPC2; K13443). Two cytoskeletal (DNAH; K10408 and KIF2; K10393) and one endocytic (CYTH; K18441) proteins also exhibited low abundances in both *Vibrio* treatments compared to the control (Fig. 6A). Six proteins showed a *Vibrio* strain-specific response. These proteins were related to endocytic (H-Hal: SNX5; K1792 and H-NJ: RAB8A; K07901) and immune pathways (H-Hal: OAS; K14216 and H-NJ: DAPP1; K12229) in both treatments (Fig. 6A).

At 60 min, we found 28 proteins related to vesicle trafficking-, bacterial infection-, and/or immune-related pathways (Fig. 6B; Table S8). The number of proteins showing high abundance decreased (15 to 11 proteins), whereas those with low abundances lightly increased (10 to 11 proteins), resulting in a similar number of proteins in both categories (Fig. 6B). More than half of the proteins in the H-H group were related to vesicle trafficking, such as a programmed cell death 6-interacting protein (PDCD6IP; K12200), a Ras-related protein (RAB8A; K07901), and a lysosomal membrane protein predicted to have scavenger receptor activity (SCARB2; K12384) (Fig. 6B). In contrast, the low abundant proteins (L-L) were largely associated with bacterial infection and immunity pathways, which included a ubiquitin C protein (UBC; K08770), a phospholipase D (PLD1; K01115), a tubulin (TUBA; K07374), a DNA-directed RNA polymerase (RPC3; K03023), and a guanine nucleotide exchange factor (RASGRP3; K12362). The only protein found to increase in abundance after exposure to Hal 281 was the cytoskeletal motor protein dynein (DNAH (1); K10408). Similarly, a dynein protein (DNAH (2); K10408) was increased in the NJ 1 treatment. The remaining proteins in the H-NJ category were histones (H2A (1) and H2A (2); K11251) and the TNF receptor (TRAF3; K03174), which showed the highest Log2 fold change (6.14) of all assessed proteins.

### Comparison of temporal dynamics in the abundance of proteins between *Vibrio* treatments

Out of the proteins that differed in abundance compared to the seawater control (Fig. 5A and B) and were related to vesicle trafficking, bacterial infection, and immunity based on KEGG identifiers (Fig. 6A and B; Table S8), 13 proteins were present both at the 30 min and the 60 min time point (Fig. 6C and D). To assess the temporal dynamics in the abundance of these proteins, four categories were created: increased abundance at both time points (I-I), decreased abundance at both time points (D-D), increased followed by decreased abundance (I-D), and decreased followed by increased abundance (D-I). Six of the 13 proteins showed a similar temporal abundance pattern in response to Hal 281 and NJ 1 (Fig. 6C and D). This *Vibrio*-specific response included proteins associated with bacterial infections (three proteins), endocytosis (two proteins), lysosome (two proteins), and autophagy/mitophagy (one protein). The remaining seven proteins showed a *Vibrio* strain-specific temporal pattern and were mainly related to endocytosis (three proteins), immune system (two proteins), and cytoskeletal/motor functions (two proteins). The endosomal protein VPS37 (K12185) as well as the two immune-related histones H2A (1) and H2A(2) (K11251) showed initially an increased abundance but were decreased at the 60 min time point (I-D) in response to Hal 281 (Fig. 6C). In contrast, the same proteins were increased in abundance at both time points in the NJ 1 treatment (I-I) (Fig. 6D). The endocytic protein SNX5 (K1792) and the cytoskeletal motor protein DNAH(1) (K10408) were increased in abundance at both time points in the Hal 281 treatment (I-I), yet were initially decreased and then increased in abundance in response to NJ 1 (D-I) (Fig. 6C and D). Interestingly, the second cytoskeletal motor protein DNAH(2) (K10408) was decreased at both time points in response to Hal 281 (D-D), while it was initially decreased and then increased in abundance in the NJ 1 treatment (D-I) (Fig. 6C and D). Lastly, the endocytic-related protein RAB8A (K07901) was initially decreased but then increased in abundance in the Hal 281 treatment, yet it was increased at both time points in response to NJ 1 (Fig. 6C and D).

## DISCUSSION

In the current study, we analyzed the cellular and proteomic response of *H. panicea* upon encounter with the sponge-isolated *Vibrio* Hal 281 and the anemone-isolated *Vibrio* NJ 1 after 30 and 60 min exposure using the combination of a recently developed *in-vivo* assay (42) with proteomics analysis. The percentage of sponge cells engaged in phagocytic activity was similar between the tested isolates. Yet, the phagocytic response differed between Hal 281 and NJ 1, both in the sponge cell types involved in phagocytosis as well as the number of vibrios incorporated per cell. Sponges exposed to the native Hal 281 *Vibrio* showed a proteomic signature that was more similar to naïve (control) sponges than to foreign NJ 1-treated sponges. Further differences between the *Vibrio* isolates in their proteomic profiles pointed to a shift from immune-related to vesicular trafficking-related proteins for the native Hal 281, whereas immune-related proteins remained increased for the foreign NJ 1.

### Choanocytes likely comprise the majority of phagocytic cells

Fluorescence-activated cell sorting revealed that more than 50% of the phagocytically active cells (i.e., with internalized vibrios) were in the size range of 5–6 µm in both *Vibrio* treatments and time points (Fig. 1; Table S3). Based on their (i) size range (*H. panicea* choanocytes: 3–7 µm [56, 57]), (ii) earlier reports of rapid incorporation of bacteria and small particles (≤2 µm) into choanocytes (e.g., references 28, 58), and (iii) the presence of a flagellum (59), we propose that these active phagocytic cells are choanocytes. We also observed cells in this size range without visible flagella (Fig. 1A). These cells may as well be choanocytes that either lost their flagellum during the cell dissociation process, or the flagellum was not noticeable due to the limited resolution obtained by fluorescence microscopy, as previously described using scanning electron microscopy (60). An alternative explanation is that these or at least a fraction of these small non-flagellated cells represent pinacocyte-like cells. Pinacocytes presumably capture large particles (>ostia diameter) by filopodial extensions and intracellularly digest them to prevent clogging of the sponge’s filtering system (61–63). The medium (7–10 µm) and big (>10 µm) phagocytically active cells (Fig. 1A) are most likely archaeocyte-like cells, given their bigger size and the presence of a large nucleus, which, in some cases, contained a distinctive nucleolus. Archaeocytes are amoeboid totipotent stem cells that move throughout the sponge mesohyl to participate in intracellular digestion and transport of food and particles between cells (64–66) but also neutralize foreign material as an immune defense system (4).

### Incorporation into phagocytic cells is *Vibrio* strain specific

Cellular processing of the native Hal 281 and the foreign NJ 1 *Vibrio* isolates appeared to be largely similar with comparable abundances of phagocytically active cells (Fig. 2D). Also, the distribution of phagocytically active cell types showed a similar temporal pattern for both *Vibrio* isolates: the percentages of small flagellated and no-flagellated cells tended to decrease from the 30 to the 60 min timepoint, whereas those of medium and big cells appeared to increase. This trend may suggest that choanocyte-like cells initially incorporate vibrios (30 min timepoint; see general proteomic response to vibrios below). Once a saturation capacity is reached (e.g., five or more *Vibrio* cells; Fig. 3), predigested vibrios are translocated to archeocyte-like cells for further processing and transport within the mesohyl (60 min timepoint). Within this cellular processing, we, however, detected some strain-specific differences. Phagocytic cells (particularly those in the small size range, 3–6 µm) had more NJ 1 incorporated per cell than Hal 281 (Fig. 3A). This strain-specific cellular response could be explained by a faster transfer of the native Hal 281 from choanocyte-like cells to archeocyte-like cells compared to the foreign NJ 1 (Fig. 3B). Alternatively, the initial incorporation of NJ 1 could be faster than that of Hal 281, or Hal 281 is evading initial degradation in choanocyte-like cells and is, therefore, transferred faster. All three options are not mutually exclusive, and such microbe-specific differences in cellular processing appear to be widespread among marine invertebrates, including sea urchin larvae (67), deep-sea mussels (68), and sea anemones (18) (Table S9).

### Proteomic response of Hal 281 is more similar to seawater bacteria than to NJ 1

The proteomic profile of *H. panicea* incubated with Hal 281 was more similar to the control (i.e., exposure to natural seawater bacterial consortium) than to the foreign *Vibrio* treatment NJ 1 with 49 of 73 and 49 of 121 DAPs being similar in the 30 min and 60 min assays, respectively (Fig. 4). This pattern could potentially suggest a mechanism for specificity driven by the distinction between known (i.e., native Hal 281 and commonly encountered seawater bacteria) and unknown (i.e., foreign NJ 1) bacteria, rather than by bacterial type (i.e., *Vibrio* vs seawater consortia). Hal 281 typically occurs in low abundances within *H. panicea* and can also be found in seawater samples collected close to sponges (69), making it more likely to be a commensal, rather than an obligatory symbiont. Since *H. panicea* is filtering approximately 1 L of seawater g dry weight^−1^ h^−1^ (70, 71), it is expected to process millions of seawater bacteria per day, including *Vibrio* Hal 281. In contrast, the NJ 1 *Vibrio* isolate occurs in high abundance in juveniles of the sea anemone *N. vectensis* and is considered a native colonizer of this species (72). Since it is typically not found in *H. panicea* environment, it is unlikely that *H. panicea* has encountered it before. However, since merely two *Vibrio* isolates have been tested in this study, it cannot be excluded that observed differences between the two isolates may simply be strain-specific and not related to recognition vs naïve encounter. Thus, additional experiments testing more native and foreign *Vibrio* strains are necessary to support this hypothesis and to avoid overgeneralization. If supported, this would, however, imply a potential for immune memory in sponges. While we interpret this distinction from the host’s perspective, alternative mechanisms employed by each *Vibrio* to evade host recognition or degradation must also be considered. For example, the mechanisms for NJ 1 to colonize *N. vectensis* may be the ones that elicit an innate immune response in a different host, in this case, *H. panicea*.

### Encounters with Hal 281 and NJ 1 predominantly elicit a general *Vibrio*-specific proteomic response

When comparing the proteomic response of the *Vibrio* isolates to the respective control treatment (i.e., directional analysis), most differentially abundant proteins (DAPs) were shared between Hal 281 and NJ 1 (Fig. 5A and B; H-H and LL). These proteins appear to constitute a generalist response of *H. panicea* to *Vibrio* encounters, irrespective of strain. This proposed generalist response features an initial (i.e., within 30 min) increase in proteins related to transferase activity and a concomitant decrease of proteins associated with nonsense-mediated mRNA decay (Fig. 5C), which may indicate posttranslational and metabolic modifications in macrophages (73, 74), together with a regulated activation of immune receptors and production of cytokines upon microbial infection (75–77). After 60 min of exposure, proteins related to lipid transport and chaperone-mediated functions are increased, whereas those associated with GTPase signaling and protein transport are decreased. Some of the DAPs were further related to vesicle trafficking, bacterial infection, and/or immunity pathways (based on KEGG annotation; Fig. 6A and B; Table S8). Also here, the vast majority of proteins represent a general *Vibrio*-specific response at the 30 min (25 of 31 proteins) as well as the 60 min (22 of 28 proteins) time point. The high abundance of proteins related to immunity and bacterial infection, such as A disintegrin and metallaprotease 10 (ADAM10) and the regulatory mTOR protein (RAPTOR) at 30 min may represent *Vibrio* recognition and the initial activation of immune components (78, 79), which are then, however, reduced to baseline levels, or below, at the 60 min time point. Conversely, lysosomal-related proteins, including the Niemann-Pick type C2 (NPC2), were initially low in abundance yet were together with endocytic-related proteins increased at 60 min. This may suggest that the encounter with a *Vibrio* elicits an initial upregulation of pathways associated with the recognition, intracellular incorporation, and elimination of a potential bacterial threat, followed by the dampening of these immune components and the activation of endosomal pathways to facilitate the sorting, transportation, and processing of the incorporated *Vibrio* cells.

### Attenuation of immune response towards Hal 281 and delayed onset of vesicle trafficking in NJ 1 suggests strain-specific responses to *Vibrio* exposure

Apart from the predominant generalist *Vibrio*-specific response, subtle but distinct strain-specific differences could be detected in the proteome of *H. panicea* after exposure to Hal 281 and NJ 1. Particularly, functions related to mitochondrial respirasome assembly were initially increased in response to Hal 281, followed by proteins associated with signal transduction, response to stimulus, cell communication, and biological regulation (Fig. 5C). In contrast, for NJ 1 we only detected an increase in biological functions related to regulation of signal transduction and cilium assembly at the 30 min time point. At this time point, proteins related to endocytic and immunity pathways were increased in both *Vibrio* treatments yet represented by different proteins (Fig. 6A). Interestingly, the endocytic protein VPS37 as well as the two immune-related histones H2A (1)/(2) were initially increased in the Hal 281 treatment but were then decreased at 60 min, whereas these three proteins were increased at both time points in response to NJ 1 (Fig. 6C and D). This may represent an initial immune response toward both vibrios, for example, by increasing the release of decondensed chromatin from phagocytic cells to capture potentially harmful vibrios (similar to neutrophil extracellular traps: NETs) (80), as indicated by the two histones [H2A (1)/(2)]. NETs consist of cell material released during cell death that traps and kills pathogens either through antimicrobial proteins or by immobilization and facilitating their incorporation into phagocytic cells (80, 81). The concomitant regulation of vesicle trafficking- and sorting-related proteins (VPS37, SNX5, and RAB8A) may further facilitate the endosomal sorting and degradation of internalized vibrios. However, this initial immune response appears to be attenuated within 60 min of Hal 281 exposure, potentially indicating recognition of the “known” *Vibrio*. Conversely, the endocytic sorting nexin SNX5 and the cytoskeletal motor dynein DNAH (1) increased in abundance at both time points in response to Hal 281, while they initially decreased and then increased in the NJ 1 treatment. Since both proteins are associated with the transfer of internalized material to endosomes for subsequent degradation (82, 83), the observed temporal pattern may indicate that Hal 281 is rapidly translocated for further processing, whereas this translocation may occur at a later time point for internalized NJ 1, which is in line with our cellular observations (Fig. 3). The increased activity of a second cytoskeletal motor protein DNAH (2), which was only increased in response to NJ 1 at 60 min, may further contribute to this transfer of accumulated vibrios.

### Conclusion

The application of the *in vivo* phagocytosis assay in the sponge *H. panicea* in combination with fluorescence microscopy and proteomics proved to be a powerful tool to investigate the internalization and processing of the native *Vibrio* Hal 281 and the foreign *Vibrio* NJ 1. Our data suggest that initially (i.e., 30 min timepoint) both strains were indiscriminately incorporated into choanocyte-like cells (Fig. 2 and 3), where they may have been recognized, as indicated by an increased abundance of proteins related to immune recognition, signaling, and elimination of a potential threat (Fig. 6A). Within 60 min, this immune response was dampened, and endosomal pathways facilitating the sorting, transportation, and processing of incorporated *Vibrio* cells were activated (Fig. 6B). Besides this predominant generalist response to *Vibrio* encounters, subtle but distinct differences occurred between the two *Vibrio* strains: for Hal 281, the initial immune response was attenuated within 60 min of exposure, and vesicular trafficking was activated (Fig. 6C), whereas the immune response toward NJ 1 remained elevated (Fig. 6D). These proteomics data are corroborated by cellular observations suggesting the fast transfer of Hal 281 from choanocyte-like cells to archaeocyte-like cells (Fig. 3A), compared to an accumulation of NJ 1 in choanocyte-like cells (Fig. 3B). This *Vibrio* strain-specific response in combination with the largely similar proteomic profile between Hal 281 and the seawater control (containing a natural consortium of seawater bacteria) (Fig. 4) may indicate a mechanism for specificity driven by the discrimination between known (i.e., native Hal 281 and commonly encountered seawater bacteria) and unknown (i.e., foreign NJ 1) bacteria, which may point toward a certain degree of immune memory in sponges.

## Data Availability

The authors confirm that the data supporting the results and conclusions of this study are accessible within its supplemental information. If further data are required, they will be made available by the authors on request. LC–MS raw data files have been deposited to the ProteomeXchange Consortium by the PRIDE partner repository with the data set identifier PXD063400.

## SUPPLEMENTAL MATERIAL

Text S1 mbio.01474-25-s0001.docx Details on the flow cytometry analysis.

Supplemental figures mbio.01474-25-s0002.pdf Figures S1-S5.

Tables S5-S8 mbio.01474-25-s0003.xlsx Proteomic analysis results, including direct pathway analysis and functional annotation of differentially abundant proteins.

Table S1 mbio.01474-25-s0004.xlsx Flow cytometry estimates.

Table S2 mbio.01474-25-s0005.xlsx FACS quantification of phagocytic sponge cells of *H. panicea* individuals used during the phagocytic assays.

Table S3 mbio.01474-25-s0006.xlsx Fluorescent microscopy counts of sponge phagocytic cells and number of *Vibrio* cells observed *H. panicea* individuals.

Table S4 mbio.01474-25-s0007.xlsx Permanova test of microscopy cell counts.

Table S9 mbio.01474-25-s0008.docx Compilation of phagocytic responses in marine invertebrates upon encounter with different bacteria and particles based on literature.

ASM does not own the copyrights to Supplemental Material that may be linked to, or accessed through, an article. The authors have granted ASM a non-exclusive, world-wide license to publish the Supplemental Material files. Please contact the corresponding author directly for reuse.
