# Supplementary material for: Combined cellular and proteomics approach suggests differential processing of a native and a foreign vibrio in the sponge Halicondria panicea
Source: mBio. 2025 Jun 27;16(8):e01474-25. doi: 10.1128/mbio.01474-25 (PMC12345144; doi:10.1128/mbio.01474-25)
Supplement: Text S1 — Details on the flow cytometry analysis. [file mbio.01474-25-s0001.docx]

**Text S1.** Estimating *H. panicea* bacterial uptake during the phagocytic assays

Water samples were taken through the incubation period (at 0 min, 2 min, 7 min, 14 min, 22 min, 30 min, 45 min, and 60 min), fixed with paraformaldehyde and glutaraldehyde in 1x PBS (final concentration 1% and 0.05%, respectively), and analyze via flow cytometry to assess bacterial uptake (i.e., filtration) by *H. panicea*. Incubations without sponges (unfiltered seawater only *Vibrio* isolates) served as controls (n = 4). The average initial concentration of the native *Vibrio* isolate Hal 281 in the seawater at the start of the 30 min and 60 min incubation was on average (± S.D. throughout the text, unless stated otherwise) approx. 1.2 x 10^5^ ± 2.2 x 10^4^ bacteria mL^-1^ and 6.6 x 10^4^ ± 8.5 x 10^3^ bacteria mL^-1^, respectively (Fig. S2 A-D, Table S1). In the 30 min incubations, the *Vibrio* concentration at T_0min_ and T_2min_ was around 1.3 to 1.5 times lower than the concentration estimated for the other time points, suggesting that the isolate Hal 281 needed around 5 to 7 min to be completely mixed in the incubation chamber (Fig. S2 A-B). In the assays that run for 60 min no mixing effect was evident (Fig. S2 C-D). The starting concentration of the isolate NJ 1 was on average 7.7 x 10^4^ ± 2.4 x 10^4^ bacteria mL^1^ in the 30 min incubations and 4.9 x 10^4^ ± 1.1 x 10^4^ in the 60 min incubations (Fig. S2 E-H). No mixing effect was observed in either of the incubation runs. Contrary to Hal 281, the concentration of the foreign *Vibrio* isolate NJ 1 did not seem to decrease either in the 30 min or the 60 min assays. Uptake rates (filtration) were not possible to estimate for either of the isolates since the flow cytometry analysis from the seawater samples was very variable along the sampled time points and sponge biological replicates.
