# Supplementary material for: Combined cellular and proteomics approach suggests differential processing of a native and a foreign vibrio in the sponge Halicondria panicea
Source: mBio. 2025 Jun 27;16(8):e01474-25. doi: 10.1128/mbio.01474-25 (PMC12345144; doi:10.1128/mbio.01474-25)
Supplement: Supplemental figures — Figures S1-S5. [file mbio.01474-25-s0002.pdf]

# Marulanda-Gomez et al. mBio
# Supplementary material

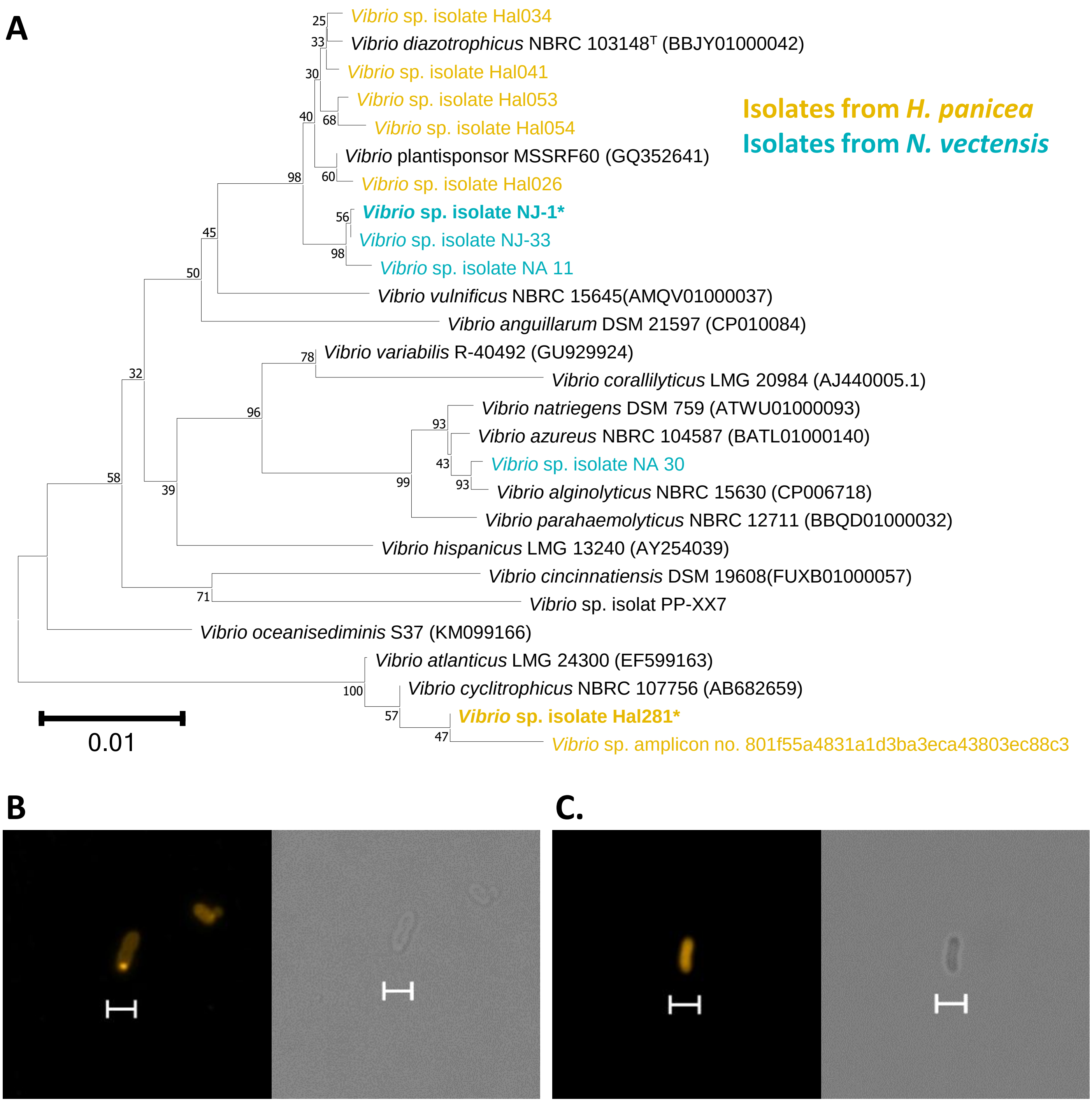


Scale: 2 µm

**Fig. S1.** Molecular and morphological comparison between the native and foreign *Vibrio* sp. Hal 281 and NJ 1, respectively. (A) Neighbor-joining phylogenetic tree of *Vibrio* strains isolated from the sponge *Halichondria panica* (yellow) and the sea anemone *Nematostella vectensis* (blue). Isolates used for the phagocytic assays are depicted in bold and with *. Environmental isolates were also included (in black) for comparison. The optimal tree is shown. The percentage of replicate trees in which the associated taxa clustered together in the bootstrap test (1000 replicates) are shown next to the branches. The tree is drawn to scale, with branch lengths in the same units as those of the evolutionary distances used to infer the phylogenetic tree. The evolutionary distances were computed using the Tamura-Nei method [3] and are in the units of the number of base substitutions per site. This analysis involved 27 nucleotide sequences based on 16S rRNA gene sequencing. Codon positions included were 1st+2nd+3rd+Noncoding. All ambiguous positions were removed for each sequence pair (pairwise deletion option). There were a total of 1546 positions in the final dataset. Evolutionary analyses were conducted in MEGA11. Fluorescence microscopy pictures of (A) Hal 281 and (B) NJ 1 stained with TAMRA. Left: TAMRA fluorescence. Right: Brightfield.

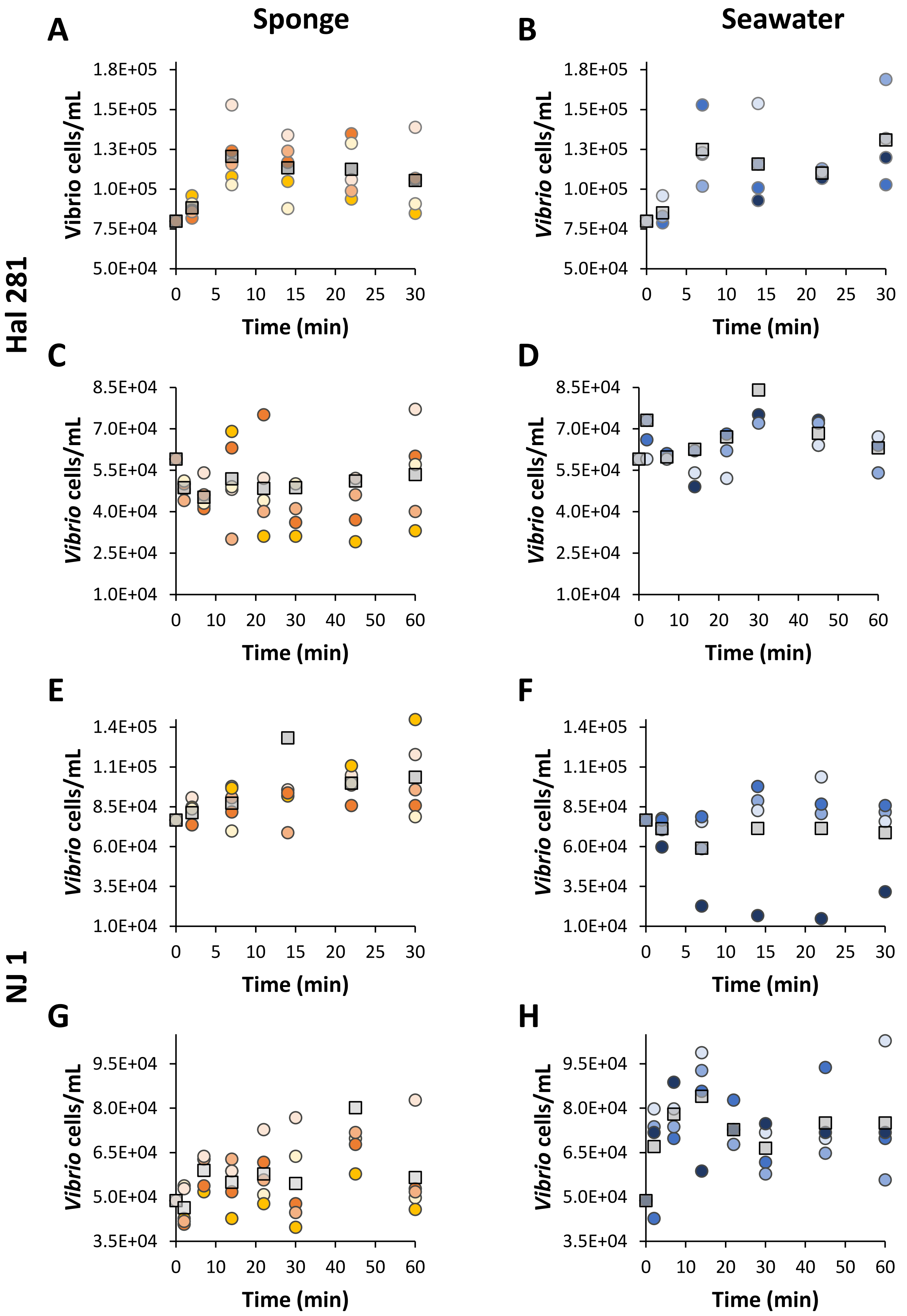


**Fig. S2.** *Vibrio* uptake by *H. panicea* individuals incubated with **A.-D.** a native (Hal 281) and **E.-H.** foreign (NJ 1) *Vibrio* isolate for 30 min and 60 min based on flow cytometry water sample analyses. Dots of the same color: biological replicates (n = 4-5 per treatment). Squares: averaged data.

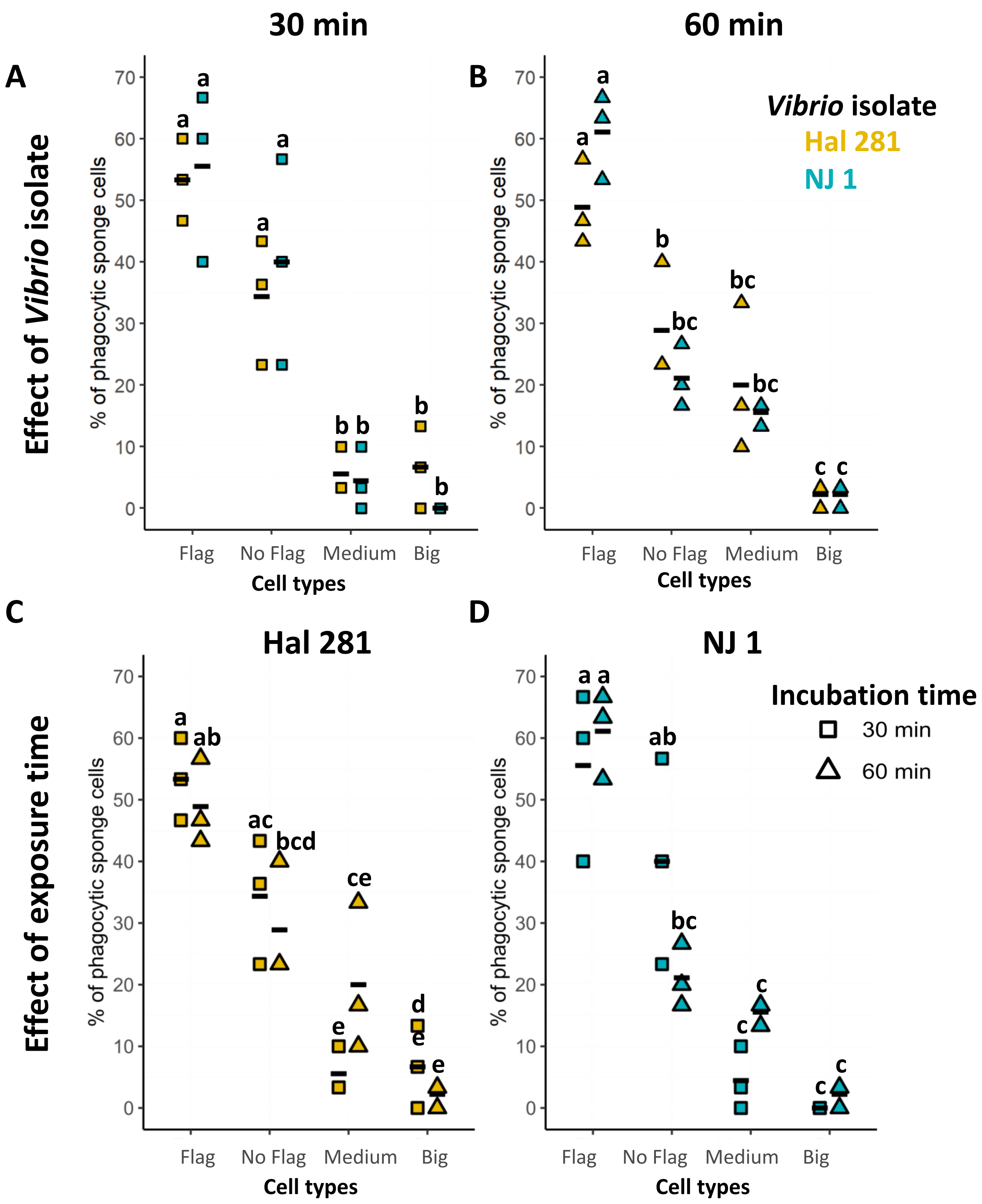


**Fig. S3.** Phagocytic cell types observed in the assays with *H. panicea.* Relative abundance of phagocytic cell types observed after 30 min and 60 min incubations with the native (Hal 281) and foreign (NJ 1) *Vibrio* isolates, based on microscopy cell counts. Bold line: average for the 3 biological replicates. Treatments marked with different letters are significantly different at a=0.05.

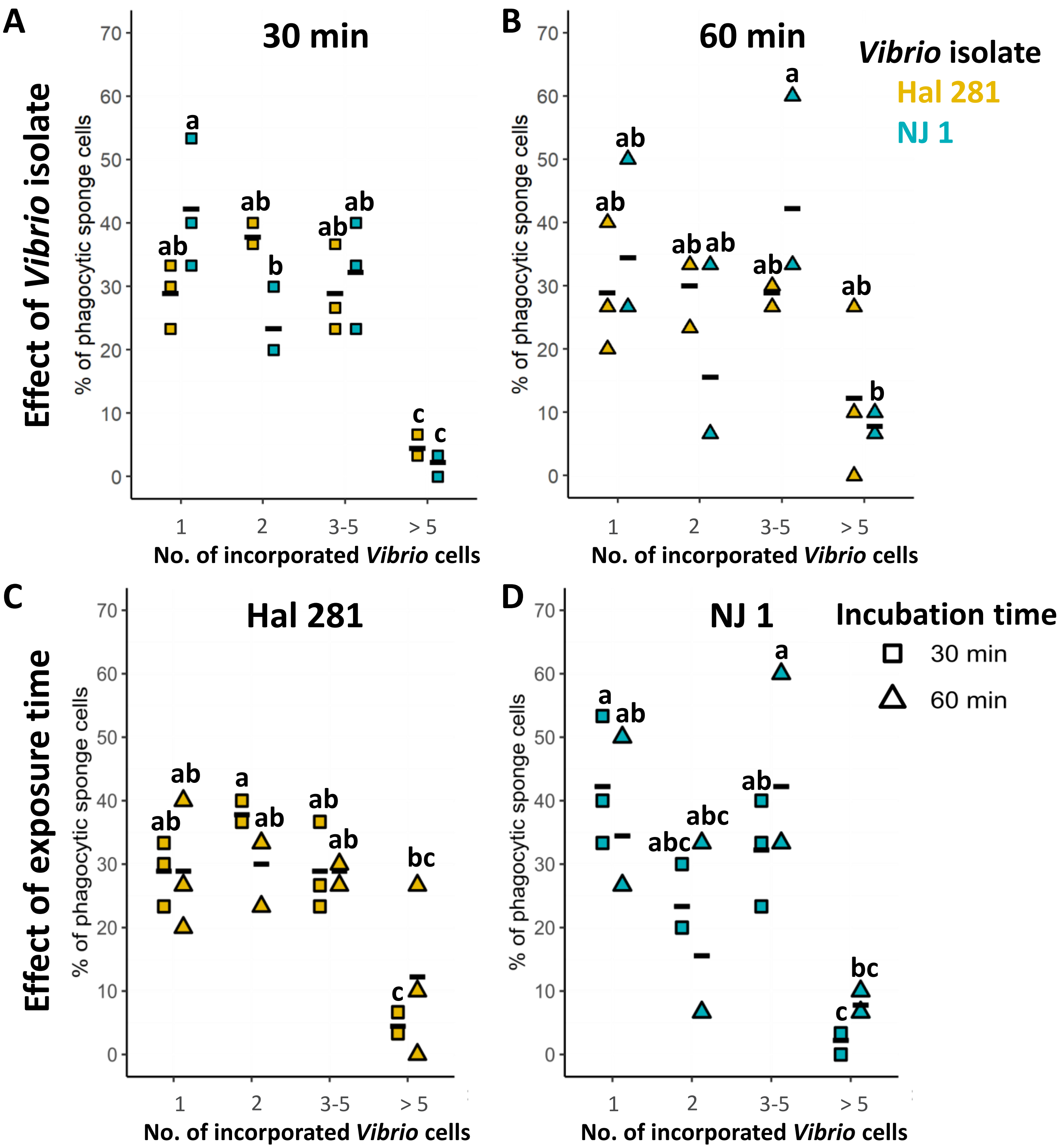


**Fig. S4.** *Vibrio* cells observed per phagocytic cell in the assays with *H. panicea.* Relative abundance of phagocytic cells per *Vibrio* category after 30 min and 60 min incubations with the native (Hal 281) and foreign (NJ 1) isolate, based on microscopy cell counts. Bold line: average for the 3 biological replicates. Treatments marked with different letters are significantly different at a=0.05.

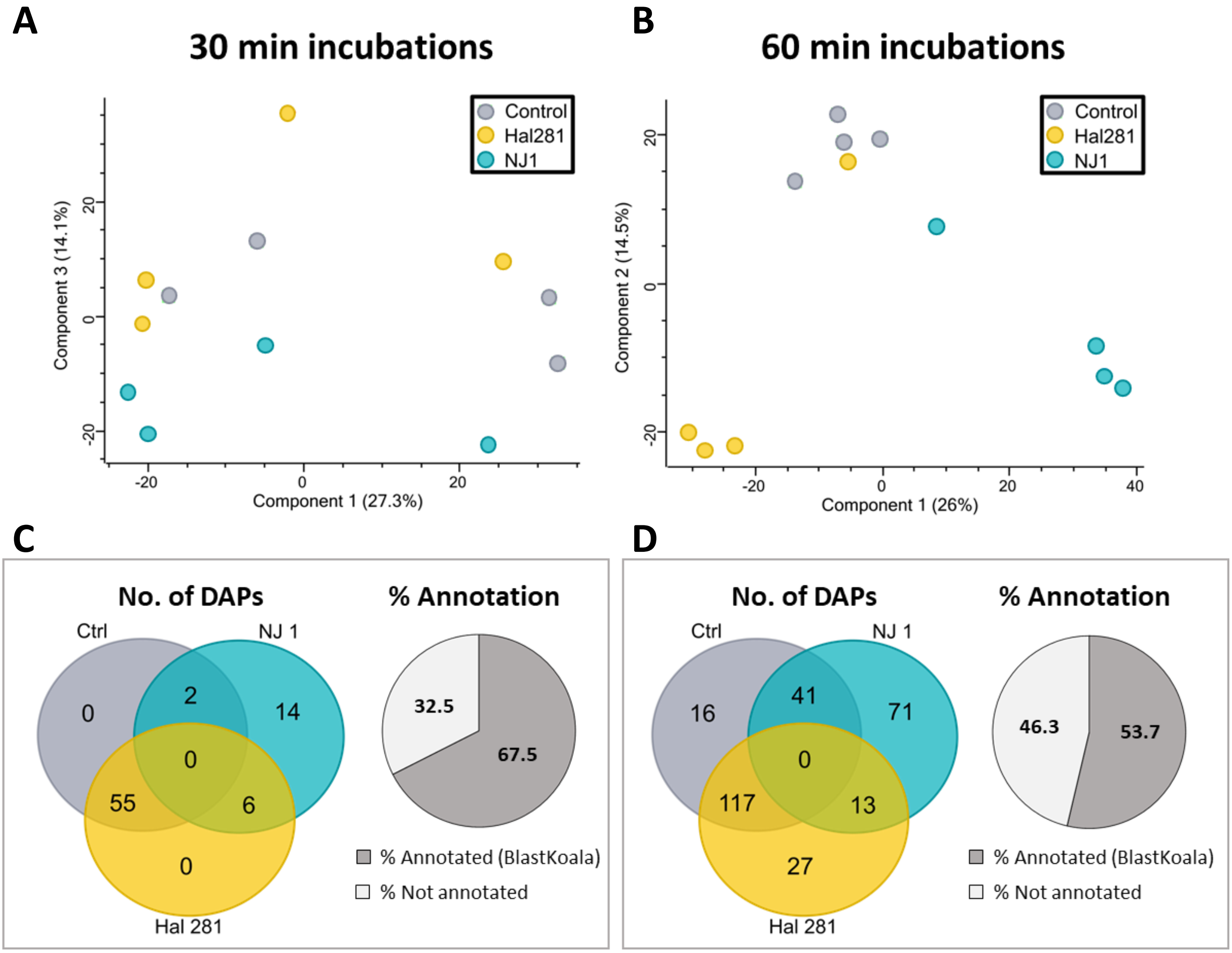


**Fig. S5.** Proteome comparison of *H. panicea* cells after 30 min (left) and 60 min (right) sponge incubations with the native and foreign *Vibrio* isolates Hal 281 and NJ 1, respectively. Sponges incubated without the addition of *Vibrio* isolates served as controls. (A) - (B) Principal component analysis showing the clustering of treatments for each time point. (C) – (D) Number of significantly differentially abundant proteins (DAPs) per treatment. Proteins were defined as differentially abundant with ANOVA permutation-based FDR = 0.05. Percentage of DAPs that could be annotated based on Blastkoala.
