# Supplementary material for: Combined cellular and proteomics approach suggests differential processing of a native and a foreign vibrio in the sponge Halicondria panicea
Source: mBio. 2025 Jun 27;16(8):e01474-25. doi: 10.1128/mbio.01474-25 (PMC12345144; doi:10.1128/mbio.01474-25)
Supplement: Table S9 — Compilation of phagocytic responses in marine invertebrates upon encounter with different bacteria and particles based on literature. [file mbio.01474-25-s0008.docx]

**Table S9.** Phagocytic response in marine invertebrates upon encounter with different bacteria and particles.

| **Organism** | **Stage** | **Particle type** | **Response** | **Reference** |
| --- | --- | --- | --- | --- |
| *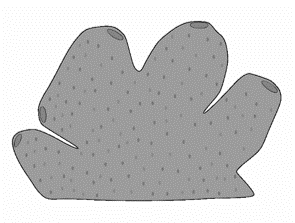 H. panicea* | Adult | Non-symbiotic microalgae (*Nannochloropsis* sp.) | Phagocytosis by choanocyte-like cells initiates within 30 min.  Algal translocation to archaeocyte-like cells for presumably digestion takes place after 30 min. Phagocytosis and algal concentration were positively related. | Marulanda-Gomez et al. 2023 |
|  |  | Non-symbiotic bacteria (*Vibrio* PPXX7) | Phagocytized primarily by choanocyte-like cells already within 30 min. |  |
|  |  | Latex beads (1 µm) | Phagocytized primarily by choanocyte-like cells already within 30 min, where they accumulated as they cannot be digested by the sponge cells. |  |
|  | Single osculum explants | Non-symbiotic bacteria (*Cyanobium bacillare*) | Phagocytized by choanocytes within approx. 40 min. | Funch et al. 2023 |
|  |  | Non-symbiotic microalgae *Rhodomonas salina* | Initially phagocytized in incurrent canals by exopinacocytes and transferred to the mesohyl by amoeboid cells for further digestion withing approx. 60 min. |  |
|  |  | Plastic beads (2 µm) | Captured by choanocytes and phagocytized by exopinacocytes on the outer sponge surface and in endopinacocytes lining the excurrent canals, and subsequently expelled into excurrent canals after approx. 60 min. |  |
|  |  | Plastic beads (10 µm) | Phagocytized by exopinacocytes on the outer sponge surface, in endopinacocytes in the mesohyl, and in endopinacocytes lining the excurrent canals, and transferred to the mesohyl by amoeboid cells. Subsequently, they were expelled into excurrent canals after approx. 95 min. |  |
| *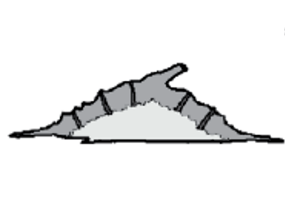 Amphimedon queenslandica* | Oscula staged juveniles | Native bacteria | Phagocytized by choanocytes and rapidly translocated to archaeocytes. | Yuen 2016 |
|  |  | Foreign bacteria | Phagocytized and digested by choanocytes. |  |
| 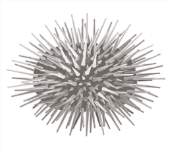 *Strongylocentrotus purpuratus*  (sea urchin) | Larvae | Non-symbiotic bacteria (*E. coli*) | Weak phagocytosis; bacteria were rarely incorporated by filopodial cells. | Ho et al. 2016 |
|  |  | Zymosan A | Rapid phagocytosis by filopodial and ovoid cells within 30 min. |  |
|  |  | Non-symbiotic bacteria (*V. diazotrophicus*) | Robust phagocytosis by filopodial cells.  Involves migration of pigment and amoeboid cells.  Around 80-90% of the bacteria are phagocytosed after 2 h. |  |
| 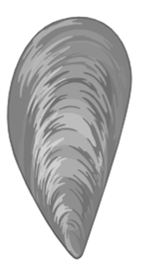 *Bathmodiolus japonicus* (deep-sea mussel) | Adult | Symbiotic bacteria (Methane-oxidizing consortia) | Phagocytized by gill cells and retained in bacteriocytes.  No acidification of bacteriocytes was observed within 24 h. | Tame et al. 2022 |
|  |  | Non-symbiotic bacteria (*E. coli* and *V. tubiashii*) | Phagocytized by gill cells and digested. Acidification of phagocytic cells started within 2 h and continued to increase after 24 h. |  |
| 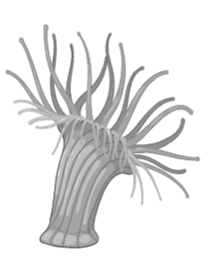 *Aiptasia* sp.  (sea anemone) | Larvae | Symbiotic microalgae (*Breviolum minutum*) | Phagocytized by endodermal cells. Involves accumulation of LAMP1 which allows symbiont intracellular persistence. | Jacobovitz et al. 2021 |
|  |  | Non-symbiotic microalgae (*N. oculata*) | Phagocytized by endodermal cells and subsequent expulsion via vomocytosis after approx. 6 h |  |
|  |  | Polystyrene beads |  |  |
|  | Adult | Non-symbiotic bacteria (*Staphylococcus aureus*) | Phagocytized by granular spheroid or ameboid cells which contained low pH vesicles, indicating the creation of phagolysosomes. | Snyder et al. 2021 |
|  |  | Carboxylated beads |  |  |

Images downloaded from Biorender or drawn by the author in Inkscape (no copyright).
